# Pathogen Challenge and Dietary Shift Alter Microbiota Composition and Activity in a Mucin-Associated *in vitro* Model of the Piglet Colon (MPigut-IVM) Simulating Weaning Transition

**DOI:** 10.3389/fmicb.2021.703421

**Published:** 2021-07-19

**Authors:** Raphaële Gresse, Frédérique Chaucheyras-Durand, Juan J. Garrido, Sylvain Denis, Angeles Jiménez-Marín, Martin Beaumont, Tom Van de Wiele, Evelyne Forano, Stéphanie Blanquet-Diot

**Affiliations:** ^1^INRAE, UMR 454 MEDIS, Université Clermont Auvergne, Clermont-Ferrand, France; ^2^Lallemand SAS, Blagnac, France; ^3^Grupo de Genómica y Mejora Animal, Departamento de Genética, Facultad de Veterinaria, Universidad de Córdoba, Córdoba, Spain; ^4^GenPhySE, INRAE, ENVT, Université de Toulouse, Castanet-Tolosan, France; ^5^Center for Microbial Ecology and Technology, Ghent University, Ghent, Belgium

**Keywords:** *in vitro* model of colonic microbiota, piglet, weaning, ETEC, intestinal cells, gene expression

## Abstract

Enterotoxigenic *Escherichia coli* (ETEC) is the principal pathogen responsible for post-weaning diarrhea in newly weaned piglets. Expansion of ETEC at weaning is thought to be the consequence of various stress factors such as transient anorexia, dietary change or increase in intestinal inflammation and permeability, but the exact mechanisms remain to be elucidated. As the use of animal experiments raise more and more ethical concerns, we used a recently developed *in vitro* model of piglet colonic microbiome and mucobiome, the MPigut-IVM, to evaluate the effects of a simulated weaning transition and pathogen challenge at weaning. Our data suggested that the tested factors impacted the composition and functionality of the MPigut-IVM microbiota. The simulation of weaning transition led to an increase in relative abundance of the *Prevotellaceae* family which was further promoted by the presence of the ETEC strain. In contrast, several beneficial families such as *Bacteroidiaceae* or *Ruminococcaceae* and gut health related short chain fatty acids like butyrate or acetate were reduced upon simulated weaning. Moreover, the incubation of MPigut-IVM filtrated effluents with porcine intestinal cell cultures showed that ETEC challenge in the *in vitro* model led to an increased expression of pro-inflammatory genes by the porcine cells. This study provides insights about the etiology of a dysbiotic microbiota in post-weaning piglets.

## Introduction

In early life, the gut microbiota is shaped by its host and by external factors, including diet (Frese et al., 2015). At weaning, piglets are exposed to social, environmental and dietary stresses engendering disruptions of the balance between intestinal microbial communities, also called dysbiosis (Gresse et al., 2017). Gut dysbiosis in post-weaning piglets is associated with a higher risk of developing infectious post-weaning diarrhea (Gresse et al., 2017), raising a major economic burden in swine industry because of the reduced growth performance and high mortality of infected animals (Amezcua et al., 2002; Fairbrother et al., 2005; Luppi et al., 2016). Additionally, the massive use of antibiotics as preventive and curative treatment increases public health concerns due to the expansion of bacteria resistance against antibiotics (Gresse et al., 2017). The major pathogenic agent responsible for post-weaning diarrhea is Enterotoxigenic *Escherichia coli* (ETEC) (Amezcua et al., 2002; Fairbrother et al., 2005; Dubreuil et al., 2016; Luppi et al., 2016; Rhouma et al., 2017). This pathotype is characterized by both the presence of fimbrial adhesins inducing cell attachment to porcine intestinal epithelial cells and secretion of enterotoxins which impact intestinal homeostasis (Dubreuil et al., 2016; Luppi et al., 2016). The most prevalent ETEC strains found in 45.1% of diarrheic post-weaning piglets harbor the fimbriae F4 (also designated K88) and secrete heat-labile toxin (LT) and heat-stable toxins (St a or b) (Fairbrother et al., 2005; Dubreuil et al., 2016; Luppi et al., 2016). If contributing factors to the progression and severity of ETEC infections such as housing conditions, early weaning, feed management, and genetic predispositions were previously identified (Madec et al., 1998; Main et al., 2004; Laine et al., 2008; Rhouma et al., 2016), the exact etiology of post-weaning diarrhea and ETEC infections remains far from understood. One hypothesis incriminates the reduced feed intake encountered by piglets at weaning which contributes to intestinal inflammation and morphology disruptions and strongly correlates with the risk of developing enteric diseases (McCracken et al., 1999; Le Dividich and Sève, 2000; Main et al., 2004; Rhouma et al., 2017). In particular, the disturbance of the mucosa intestinal environment and its associated microbiota could promote the expansion of opportunistic pathogens such as *Enterobacteriaceae* and increase the susceptibility toward bacteria and their toxins (Gresse et al., 2017). At the end of the weaning-induced feed deprivation period, the ingestion of plant-based derived solid feed further remodels the composition of the gut microbiota that was adapted to maternal milk during the suckling period (Frese et al., 2015). Understanding the origin of post-weaning diarrhea is challenging since the mechanisms involved are very complex and probably caused by nutrition and both host and microbe-derived factors. *In vitro* models of the piglet intestine including gut microbiota are adequate tools to remove host influence and thus exclusively evaluate factors impacting or influenced by commensal microbes. Especially, the use of such *in vitro* techniques offers advantageous conditions when pathogenic strains are involved due to more standardized conditions, good reproducibility and ethical reasons (Payne et al., 2012). Hitherto, the PigutIVM (Piglet Gut *In vitro* Model) and the BABY-SPIME (Baby Simulator of Pig Intestinal Microbial Ecosystem) were the only developed *in vitro* models mimicking the specific physicochemical and microbial conditions encountered in the colon of piglets (Fleury et al., 2017; Dufourny et al., 2019). However, the recently designed MPigut-IVM (Mucin associated Piglet Gut *In vitro* Model) brought the unique feature of reproducing the mucus-associated microbiota of piglet colon using specifically developed mucin beads. In a previous study using the MPigut-IVM, a 48 h feed deprivation stress remodeled piglet gut microbiota composition and functionality (Gresse et al., 2021).

In this study, we used the MPigut-IVM to evaluate the impact of a dietary change on the gut microbiota of 4-week-old piglets after a 48 h feed deprivation period. The MPigut-IVM was then exposed to an ETEC strain isolated from diarrheic piglets to study the interactions between the pathogen and gut microbiota. Finally, to unravel the consequences of microbiota perturbation on the host epithelium metabolism, filtrated effluents of control and ETEC-inoculated MPigut-IVM bioreactors were incubated with a porcine cell line.

## Materials and Methods

### Fecal Sample Collection and Treatments

All animals were housed in a conventional pig farm located in the Haute-Loire area of the Auvergne-Rhône-Alpes region in France. Piglets remained with their mother and siblings during the suckling period. In addition to sow milk, piglets received water and pre-weaning diet *ad libitum*. None of the piglets had signs of enteric or metabolic disturbances. The animals did not receive any antibiotic in the 27 days prior to the day of fecal collection. As freezing process showed to affect bacterial abundances in pig feces (Metzler-Zebeli et al., 2016), fecal samples from six 4-weeks old healthy male unweaned suckling piglets (Landrace × Large White) were collected directly from the pig while holding using sterile bottles and preserved under anaerobic conditions using GENbag anaer gas pack systems (bioMérieux, Marcy-l’Etoile, France) during transport to laboratory where they were immediately processed upon their arrival.

### MPigut-IVM Parameters

Five hundred milliliters MiniBio bioreactors (Applikon Biotechnology, Delft, Netherlands) equipped with stirrers, ports and probes and inoculated with fecal samples from piglets were prepared as previously described (Gresse et al., 2021). Briefly, 150 mL of fecal suspension prepared in an anaerobic chamber were added in each bioreactor containing 150 mL of nutritive medium (see below), previously reduced by flushing with O_2_-free N_2_ gas. Ten minutes after inoculation and during the fermentation course, flushing was stopped and anaerobic conditions were maintained exclusively by the activity of the resident microbiota and by ensuring the airtightness of the system. The temperature of the fermentation was set up to 39°C, pH was maintained to a physiological value of 6.0, and redox potential was constantly measured. The fermentation medium was stirred at a constant speed of 300 rpm. After a 24 h-batch fermentation period, nutritive medium was continuously introduced at a flow rate of 0.17 mL/min except during the feed deprivation period (see below). The fermentation medium volume was maintained at 200 mL using a drainage pump controlled by a level sensor. Indeed, the system ensured a retention time of 18 h to mimic the colonic transit time of 4-week old piglets (Wilson and Leibholz, 1981). Anaerobic conditions and gas composition were checked every day by analyzing N_2_, O_2_, CO_2_, CH_4_, and H_2_ present in the atmospheric phase of the bioreactors using a 490 Micro gas chromatograph (Agilent Technologies, Inc., Santa Clara, CA, United States) equipped with two columns, Molecular Sieve 5A and PoraPlot U, coupled with TCD detectors. Argon was used as gas carrier.

### Composition of MPigut-IVM Nutritive Media

**FIGURE 1:**
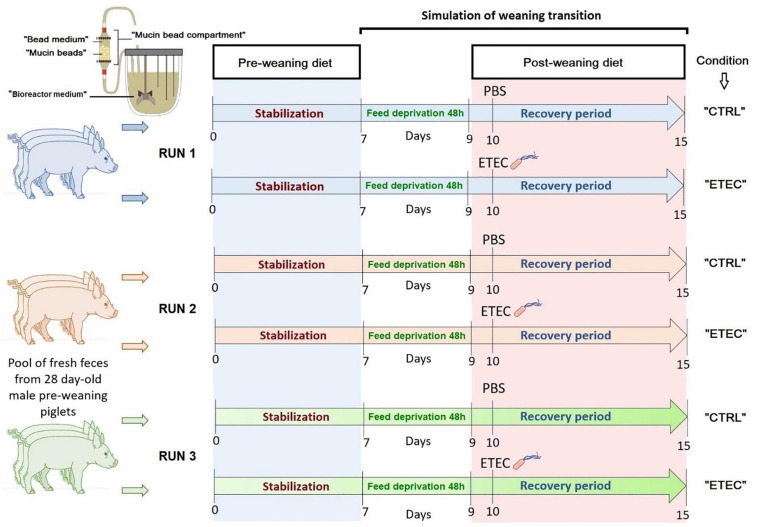
Experimental design of the *in vitro* fermentations and denomination of the conditions studied in the MPigut-IVM.

Two nutritive media were used during *in vitro* fermentative procedure (see Figure 1). A pre-weaning diet was given during the first 7 days of fermentation (Stabilization period, Figure 1). Its formula was elaborated as previously described (Gresse et al., 2021) and considered as a digested pre-weaning diet (Supplementary Table 1). Following the 48 h feed deprivation period, which was simulated by stopping the nutrient supply to MPigut-IVM bioreactors, corn meal, potato protein and a higher concentration of soy proteins were included in the diet whereas milk derived proteins and products were reduced to simulate a post-weaning diet as commonly fed to piglets (Supplementary Table 1).

### Mucin Bead Production and Compartment

Mucin beads were prepared as previously described (Gresse et al., 2021). At the beginning of fermentation, 350 ± 20 mucin beads were introduced into their specific glass compartment. The latter was maintained at 39°C in a water-bath and the fermentative medium continuously flowed through it (re-circulating loop). Mucin beads were completely replaced every 48 h to ensure a continuous availability of mucin adherent surfaces. During the time of bead replacement, the medium of the bead compartment was kept under CO_2_ flushing to avoid oxygenation.

### *In vitro* Fermentation Procedures and Sampling

The first 7 days of the fermentation procedures represented the stabilization period and corresponded to the appropriate time to achieve stabilization of microbiota composition, diversity and activity inside the MPigut-IVM (Gresse et al., 2021). At day 7, the flow of nutritive medium was interrupted during 48 h to simulate feed deprivation observed at weaning. At day 9, the flow of nutritive medium restarted with a simulated post-weaning diet. Taken together the feed deprivation stress and the dietary change applied during the recovery period aimed to simulate weaning transition (Figure 1). Experiments were designed as presented on Figure 1, three independent runs of fermentation were performed with different pools of piglet fecal samples. Samples from the bioreactor medium were collected on day 7 (before the start of the feed deprivation period), day 9 (right at the end of the feed deprivation), and days 10, 10.5, 11, 12, and 15 (corresponding to the recovery period). Mucin beads and bead medium from the bead compartment were collected on days 7, 9, 11, and 15. Samples from the bioreactor medium and bead medium were centrifuged (4°C, 10,000 *g*, 45 min). Pellets and supernatants were stored until analysis at −20 and −80°C, respectively. After collection, mucin beads were gently washed three times in sterile 1X PBS and stored at −20°C.

### ETEC Strain, Culture and Challenge Conditions

The ETEC Ec105 strain (F4, Stb+, East1+, LT+) was isolated from a diarrheic piglet (Dr. J. J. Garrido, Department of Animal Genetics, University of Córdoba, Spain). Prior to ETEC challenge in the *in vitro* model, bacteria were grown until OD_600__nm_ = 0.6 in Luria Bertani (LB) broth (BD Difco, NJ, United States), at 39°C to be consistent with the temperature of the MPigut-IVM (body temperature of piglets). The bacterial culture was then centrifuged (4°C, 10,000 *g*, 15 min) and the pellet was rinsed using sterile PBS 1X (Phosphate Buffered Saline, Sigma-Aldrich), resuspended in 1 mL of sterile PBS and inoculated to the bioreactor medium of the “ETEC” condition on day 10 (see Figure 1) at a final concentration of 10^7^ CFU/mL of fermentation medium. The control condition, referred as “CTRL,” received 1 mL of sterile PBS 1X at the same time.

### PMA Treatments for qPCR

Samples from the bioreactor medium and bead medium were collected in duplicate at all time points. They were stained with 50 μM PMAxx (Interchim, Montluçon, France) as described by Roussel et al. (2018) to avoid PCR amplification of DNA from dead cells. The stained samples were incubated for 5 min in the dark at room temperature, under shaking (100 rpm). After incubation, samples were exposed to the blue light PMA-Lite LED Photolysis (Interchim, Montluçon, France) during 15 min to activate the PMAxx dye. Samples were then centrifuged (4,400 *g*, 4°C, 45 min). Pellets were washed twice with milli-Q water and stored at −20°C until DNA extraction.

### DNA Extraction From MPigut-IVM Samples

Total DNA was extracted from all samples using the Quick-DNA Fecal/Soil Microbe Miniprep Kit (Zymo Research, Irvine, CA, United States) according to the manufacturer’s instructions. The quality of the eluted DNA was assessed by agarose gel electrophoresis. Extracts were quantified using the Qubit dsDNA Broad Range Assay Kit (Invitrogen, Carlsbad, CA, United States) with a Qubit 2.0 Fluorometer (Invitrogen, Carlsbad, CA, United States). Samples were stored at −20°C prior to analyses.

### Microbial Quantification by qPCR

The list of primer pairs and their optimal conditions used for quantitative PCR of total bacteria, Methanogenic archaea and *Escherichia*/*Shigella* group are presented in Supplementary Table 2 (Huijsdens et al., 2002; Yu et al., 2005; Ohene-Adjei et al., 2008). Standard curves assessment was performed as specified in Gresse et al. (2021). Real-time PCR assays were performed on a Rotor-Gene Q (Qiagen, Venlo, Netherlands) in 20 μL reactions with QuantiFast SYBR GREEN master mix (Qiagen, Venlo, Netherlands) or TaqMan Fast Advanced Master mix (Applied Biosystems, Foster City, CA, United States) with the addition of each primer at their optimal concentration (Supplementary Table 2). The 16S rDNA genes were amplified using the following program: 2 min denaturation at 95°C and 10 min denaturation at 95°C; 40 and 45 cycles of 20 s at 95°C and 60 s elongation and extension at the optimum annealing temperature, and when performing SYBR GREEN based assay, a melting curve step was performed from 60°C to 95°C. Each reaction was run in duplicate. The melting curves of PCR amplicons from SYBR GREEN based assays were checked to ensure primer specificity. The 16S rDNA gene copy number was calculated using the formula: copy number/μl = (C/X)^∗^0.912.1012 with C: DNA concentration measured (ng/μL) and X: PCR fragment length (bp/copy) and diluted in 10-fold dilution series to be used as qPCR standards. Efficiency of the qPCR for each target varied between 95 and 105% with a slope from −3.0 to −3.4 and a regression coefficient above 0.95, which was in accordance with the MIQE guidelines (Bustin et al., 2009). Ten-fold dilutions series of DNA extracted from the ETEC Ec105 pure culture stained or not with PMA were used to control the reliability of the PMA treatment. A sample from the same bacterial pure culture was subjected to a lethal treatment (95°C, 15 min) and stained or not with PMA and used as a negative control for PMA-qPCR. The survival of the ETEC strain was monitored from day 10, i.e., the time of inoculation, to day 15 in the bioreactor medium, bead medium and on mucin beads *via* the quantification of the labile enterotoxin (LT) gene [Supplementary Table 3, references (Ngeleka et al., 2003; Rahman et al., 2006; Madoroba et al., 2009; Nicklasson et al., 2012; Roussel, 2019)]. After log transformation of the data, a mixed-model one-way ANOVA (lmer and ANOVA functions) with time point (days of fermentation) and ETEC treatment as fixed effects and fermentation experiment as a random effect was used to compare the number of 16S gene copy per g of samples between days of fermentation using the R packages lme4 package version 1.1.21 and car package version 3.0-6. The means of each group were compared pairwise with the lsmeans package (version 2.30-0) with the Tukey correction.

### MiSeq 16S rDNA Sequencing and Bioinformatic Analysis

The DNA concentration of all samples was measured using the Qubit dsDNA High Sensitivity Assay Kit (Invitrogen, Carlsbad, CA, United States) with a Qubit 2.0 Fluorometer (Invitrogen, Carlsbad, CA, United States) and diluted to 2 ng/μL prior to PCR amplification. The Bacterial V3–V4 region of 16S rDNA and the Archaeal 16S rDNA were, respectively, amplified with primers 357F 5′-CCTACGGGNGGCWGCAG-3′ (Yu et al., 2005) and 805R 5′-GACTACHVGGGTATCTAATCC-3′ (Lane et al., 1985) and primers 349F 5′-GYGCASCAGKCGMGAAW-3′ and 806R 5′-GGACTACVSGGGTATCTAAT -3′ (Ohene-Adjei et al., 2008). Amplicons were generated using a Fluidigm Access Array followed by high-throughput sequencing on an Illumina MiSeq system (Illumina, San Diego, CA, United States) performed at the Carver Biotechnology Center of the University of Illinois (Urbana, IL, United States). The demultiplexed paired end Illumina sequence reads in the FastQ format were uploaded into the Galaxy instance (v.2.3.0) of the Genotoul bioinformatics platform^1^ to be used in the FROGS (Find Rapidly OTU with Galaxy Solution) pipeline (Escudié et al., 2018). During the FROGS pre-process, sequences were depleted of barcode and the sequences with a non-appropriate length or containing ambiguous bases were removed. Next, reads were clustered into *de novo* operational taxonomic units (OTUs) using SWARM algorithm (Mahé et al., 2014) with, at first, a denoising step to build very fine cluster using the minimal distance equal to 1 and, secondly, with an aggregation distance equal to 3. Chimeras were then detected and removed with VSEARCH (Rognes et al., 2016). Additionally, filters were applied to the OTUs in order to remove singletons (Bokulich et al., 2013; Auer et al., 2017). The OTUs selected were taxonomically assigned using the Silva release 132 reference database (Quast et al., 2013).

### Statistical Analysis of Sequencing Data

The Illumina MiSeq run generated a total of 8,107,484 and 1,467,731 high quality sequences, respectively, for the V3–V4 and archaeal sets of primers. Removal of PhiX control reads, removal of chimeras and filtering of singletons lead to a number of 51,703 ± 13,520 sequences for V3–V4 primers and 5,377 ± 4,645 sequences for archaeal primers per sample. To avoid any bias, samples containing less than 500 sequences after abundance filtering were removed from the dataset. Statistical analysis was processed using the RStudio software version 1.0 (with R software version 3.5.1, R Development Core Team)^2^. OTU structure and composition analyses were performed using the phyloseq R package version 1.30.0 (McMurdie and Holmes, 2013). Visualization of data was performed using the ggplot2 R package version 3.2.1. Prior to alpha and beta diversity calculations, rarefaction using the transform count methods was applied to the dataset. The following alpha diversity indices were calculated: number of observed OTU phylogenic diversity and Shannon index. Statistical differences in Bray Curtis distance between the mucin beads, bead medium and the bioreactor medium and between the pre and post-weaning diet were tested using a multi-analysis of variance (MANOVA) performed with ADONIS using the vegan R package with 9999 permutations and represented by principal coordinate analysis (PCoA) plots. The relative abundances of bacterial groups were log transformed prior to univariate statistical analyses. All univariate statistical analyses were performed using linear mixed-models (lme4 package version 1.1.21) with time point (days of fermentation) and ETEC treatment as fixed effects and fermentation experiment as a random effect. Analysis of variance tables was calculated with the car package (version 3.0.6). The means of each group were compared pairwise with the lsmeans package (version 2.30-0) with the Tukey correction (Supplementary Table 4). Statistical comparisons of samples from the recovery phase of the fermentation containing or not the ETEC Ec105 strain were also performed using the Wald test of the DESeq2 R package version 1.26.0 at the genus level. In all statistical analyses, only *P*-values below 0.05 were considered as significant.

### RNA Isolation of MPigut-IVM Samples

Total RNAs from bioreactor medium, mucin beads, and bead medium were extracted using Trizol reagent (Invitrogen, Thermo Fisher Scientific, Waltham, MA, United States) as described by Comtet-Marre et al. (2017). DNAse treatment with the rDNAse Set (Macherey-Nagel, Hśrdt, France) was performed to remove any contamination of genomic DNA according to the manufacturer’s instructions. The integrity of few samples representative from the whole set was assessed using the Agilent 2100 Bioanalyzer using RNA Nano Chip (Agilent Technologies, Inc., Santa Clara, CA, United States) to ensure sufficient quality for RT-qPCR. Quantity and purity of RNAs were measured using the Nanodrop One (Thermo Fisher Scientific, Waltham, MA, United States) and RNAs were stored at −80°C until cDNA synthesis.

### RT-qPCR of ETEC Virulence Genes

First, 1 μg of RNA per sample was reverse transcribed into complementary DNA (cDNA) with the SuperScript IV Reverse Transcriptase kit (Invitrogen, Thermo Fisher Scientific, Waltham, MA, United States) in conformity with the manufacturer’s instructions. QPCR was performed on the cDNAs as outlined in the section above. Primers and conditions used for qPCR on cDNAs are listed in Supplementary Table 3. cDNAs and DNA samples from the ETEC Ec105 pure culture and from the MPigut-IVM challenged with ETEC were used as a positive control. The comparative E−ΔΔCt method was applied to calculate the relative fold changes in the expression of ETEC virulence genes in the samples from the MPigut-IVM. The BestKeeper excel-based tool (Pfaffl et al., 2004) was used to determine the geometric means of the three quantified reference genes, arcA, gapA, and rpos considered for normalization. Primer efficiency was determined using 10-fold dilution series of a set of samples representative from mucin beads, bead medium, and bioreactor medium. The efficiency was calculated from the slope of the standard curves using the following equation *E* = 10 (−1/slope), where E corresponds to high/acceptable amplification efficiency equals to 90–110%.

### Quantification of Short Chain Fatty Acids (SCFAs) by Gas Chromatography

The SCFAs were quantified in the bioreactor medium and bead medium by gas chromatography. Eight hundred microliters of supernatants from bioreactor medium and bead medium were mixed with 500 μL of 0.4% (w:v) crotonic acid and 2% (w:v) metaphosphoric acid solutions. This mixture was centrifuged and the supernatant obtained was injected into a PerkinElmer Clarus 580 gas chromatograph (Waltham, MA, United States) for quantification of SCFAs. A mixed-model one-way ANOVA (lmer and ANOVA functions) with time point and ETEC treatment (days of fermentation) as fixed effects and fermentation experiment as a random effect was used to compare the concentration of the main SCFAs between days of fermentation using the R packages lme4 and car.

### Metabolome Analysis by 1H Nuclear Magnetic Resonance (NMR)

Supernatants of mucin bead medium collected on days 7 and 11 were used for metabolomic profiling using NMR spectroscopy. After two centrifugation steps (18,000 *g*, 4°C, 10 min) to remove particles, 50 μL of supernatant were mixed with 600 μL of buffer composed of sodium phosphate 0.2 M, pH 7.4, trimethylsilylpropanoic acid 1 mmol/L, 80% deuterated water, and 20% water. Spectra acquisition, processing, and metabolite identifications were performed as described previously in the MetaboHUB MetaToul-AXIOM metabolomics platform (Beaumont et al., 2020). The list of metabolites identified in the bead medium is presented in Supplementary Table 5.

Statistical analysis for NMR metabolomics was performed using the R software (version 3.5.1). Partial-least square discriminant analysis (PLS-DA) was performed with mixOmics package (Rohart et al., 2017). Metabolite relative concentration was used as variable matrix (X). Groups (Day 7, Day 11 CTRL, Day 11 ETEC) were used as predictors (Y) and time-repeated measurement were considered by using a multilevel approach. Univariate statistical analysis was also performed on each metabolite relative concentration with the R packages lme4 and car. A mixed-model one-way ANOVA (lmer and ANOVA functions) with group (Day 7, Day 11 CTRL, Day 11 ETEC) as a fixed effect and fermentation experiment as a random effect was used. A *post hoc* test was used to compare the mean relative concentrations with Tukey correction. *P*-values were corrected for multiple testing (false discovery rate).

### Incubation of MPigut-IVM Effluents on the IPI Porcine Cell Line

#### IPI-2I Cell Culture

The IPI-2I cell line is derived from the ileum of an adult male pig and was immortalized by transfection with an SV40 plasmid (pSV3-neo) (Kaeffer et al., 1993). IPI-2I cells were maintained in Dulbecco’s Modified Eagle Medium (DMEM)/Ham’s F-12 (1:1) medium (Invitrogen Life Technologies, Carlsbad, CA, United States) supplemented with 10% Fetal Calf Serum (FCS, PAA Laboratories GmbH, Austria) and 4 mM L-glutamine (Sigma, St. Louis, MO, United States). Cells were seeded onto 48-well tissue culture plates at 25,000 cell/well in a volume of 200 μL and grown 24 h in an atmosphere of 5% CO_2_ at 37°C to allow for confluency for the day of experiment.

#### Exposure of IPI-2I Cells to MPigut-IVM Effluents

Supernatants from bioreactor and bead medium at days 7, 9, 11, 13, and 15 were filtered using 0.2 μm sterile Minisart syringe filters (Sartorius, Göttingen, Germany) and 30 times diluted with DMEM (10% Fetal Calf Serum and 4 mM L-glutamine). A thirty-fold dilution of each sample was established as the best compromise following preliminary tests estimating the survival of IPI-2I cells exposed to dilution series of MPigut-IVM supernatants. After 2 h of incubation with the 30 fold diluted supernatants, the viability of cells was comprised between 70 and 100% with a mean of 86.5% (*n* = 8, data not shown). The diluted samples were added in duplicate to confluent monolayers of IPI-2I cells in 48-well plates, as described above. Plates were incubated for 2 h at 37°C, 5% CO_2_. Then, the supernatants were removed and IPI-2I cells were lysed by addition of 500 μL of NucleoZOL (Macherey-Nagel, Hœrdt, France). Cell lysates were stored at −80°C prior to RNA isolation.

#### RNA Isolation From IPI-2I Lysates

Total cellular RNA was extracted from IPI-2I lysed cells following the guidelines provided by the NucleoZOL user manual (Macherey-Nagel, Hœrdt, France). The TURBO DNA-free^TM^ kit (Applied Biosystems, Foster City, CA, United States) was used according to the manufacturer’s instructions to prevent DNA contamination. Purity and quality of the RNA extracts were controlled on 1% agarose gels. RNAs were then quantified using a Nanodrop 1000 spectrophotometer (Thermo Fisher Scientific, Waltham, MA, United States) using an optical density of 260 nm.

#### RT-qPCR on IPI-2I RNA Extracts

Reverse transcription was performed using the qScript cDNA Synthesis Kit (Quantabio, Beverly, MA, United States). Briefly, 350 ng of RNA per sample were added to 5 μL of sScript Reaction Mix (5x) and 1 μL of qScript Reverse Transcriptase in a final volume of 15 μL. The reverse transcription mix was successively incubated 5 min at 22°C, 30 min at 42°C, and 5 min at 85°C. The synthetized cDNAs were stored at −20°C until used. The targeted genes are listed in Supplementary Table 5 (Mariani et al., 2009). Quantifications were carried out in triplicate for each cDNA using a QuantStudio^TM^ 12K Flex Real-Time PCR system (Applied Biosystems, Foster City, CA, United States). The cyclophilin A and β-actin genes were used as reference genes. PCR reactions were carried out in 96 well plates using 3 μL of 5x HOT FIREPol^®^ EvaGreen^®^ qPCR Mix Plus (ROX) (Solis BioDyne, Tartu, Estonia), 0.4 μL of forward and reverse primer, 9.2 μL of milli-Q water, and 2 μL of cDNA. Tenfold dilution series of each primer pair were used as standard curves to determine primer efficiencies. Real time PCR efficiencies were calculated according to the equation: *E* = 10 (−1/slope). The appropriate reference gene and the Log2 fold change of each gene, compared with the IPI-2I cells which had not been exposed to bead medium supernatants, were determined by GenEx software^3^. A mixed-model one-way ANOVA (lmer and ANOVA functions) with time point (days of fermentation) and ETEC treatment as fixed effects and fermentation number as a random effect was used to compare the significance between gene expression profile of the IPI-2I cells which were exposed to bead medium supernatants containing or not the ETEC Ec105 strain using the R packages lme4 and car. In all statistical analyses, only *P*-values below 0.05 were considered as significant.

## Results

Analyses of the microbial profile and SCFA proportions of the three pooled fecal inocula for the runs #1, 2, and 3 are available in Supplementary Figure 1. Redox potential was followed throughout the whole fermentation runs and was representative of a feed deprivation stress by displaying an important increase during this period such as detailed in Gresse et al. (2021) (data not shown). Gas composition was also monitored every day during the fermentation runs. At the end of the stabilization period [Day 7, the mean relative proportions of H_2_, O_2_, CO_2_, N_2_, and CH_4_ for the six bioreactors were, respectively, 2.1 ± 1.2, 0.3 ± 0.1, 73.7 ± 0.4, 5.9 ± 0.8, and 6.8 ± 1.1% (data not shown)].

### Simulation of Weaning Transition Affects the Metabolic Activity of the MPigut-IVM Microbiota

**FIGURE 2:**
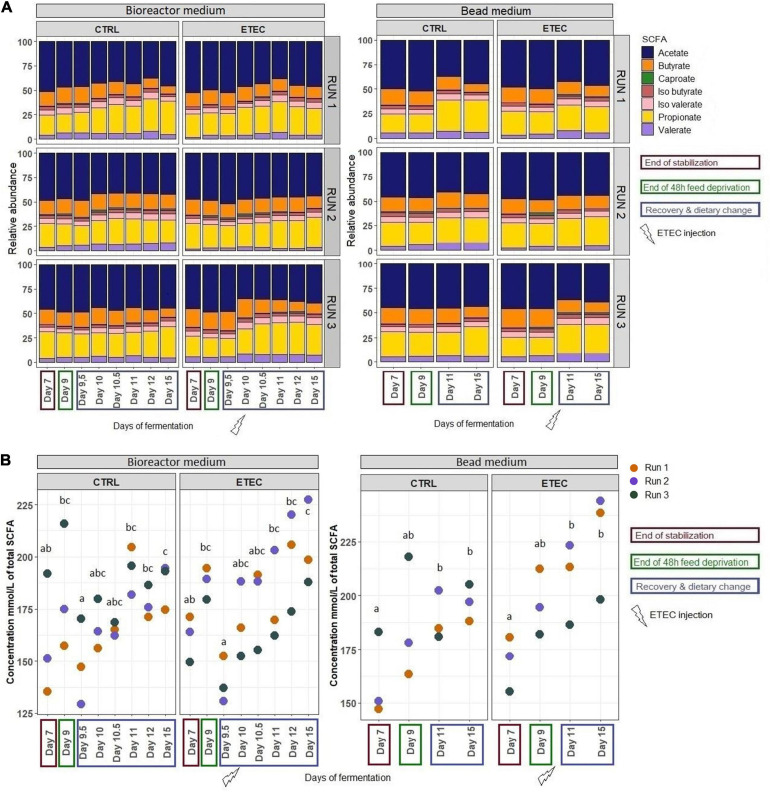
Short chain fatty acids (SCFA) relative abundances **(A)** and Evolution of the total concentration of SCFAs **(B)** produced by fermentation activity of the microbiota inhabiting the MPigut-IVM in the CTRL and ETEC conditions for the runs #1, 2, and 3. Groups (days) associated with different letters are significantly different (*P* < 0.05).

In the bioreactor medium of the CTRL group, propionate and caproate proportions increased while acetate, isovalerate and butyrate proportions decreased from day 10 to 15 compared to day 7 (Figure 2A). In the mucin bead medium of the CTRL group, the proportions of propionate, isovalerate and valerate increased while the proportions of acetate and butyrate decreased from day 11 to 15 compared to day 7 (Figure 2A). The total concentration of SCFA significantly (*P* < 0.05) increased between day 9.5 and 15 both in the bioreactor medium and the bead medium (Figure 2B).

**FIGURE 3:**
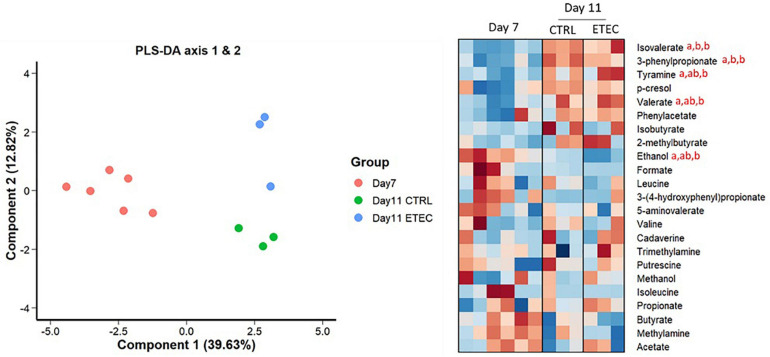
NMR-based metabolomics analysis of mucin bead medium. **Left panel:** Individual plot of PLS-DA. **Right panel:** Heatmap representing the relative concentrations of all identified metabolites (rows) in individual samples (columns). The color represents the Z-scores (row-scaled relative concentration) from low (blue) to high values (red). The days sharing the same letters are not significantly different from each other (*p* > 0.05) whatever the conditions.

Nuclear magnetic resonance-based metabolomics revealed a strong modification of the mucin bead medium metabolome between day 7 and day 11 in the CTRL group (Figure 3). The relative concentration of isovalerate and 3-phenylpropionate increased significantly after the simulation of weaning transition (day 11 CTRL).

### Simulation of Weaning Transition Impacts MPigut-IVM Microbiota Composition

**FIGURE 4:**
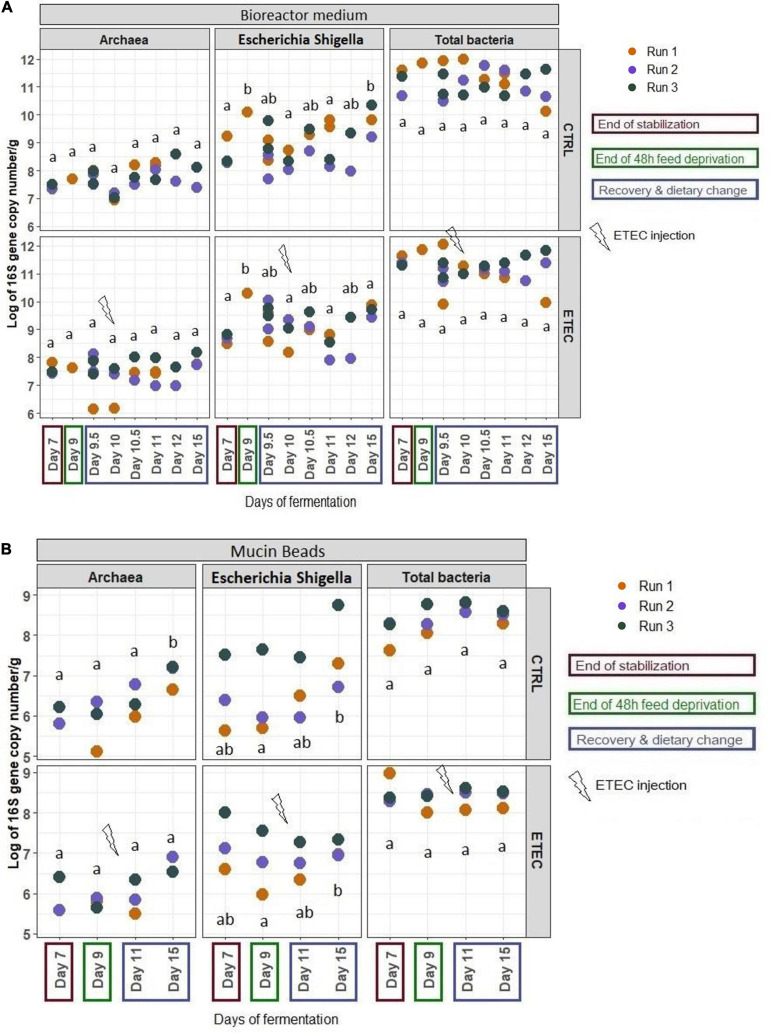
Q -PCR quantification of total bacteria, Escherichia/Shigella and methanogenic archaea populations in the bioreactor medium **(A)** and the mucin beads **(B)** of the MPigut-IVM for the runs #1, 2, and 3 in the CTRL and ETEC conditions. The days sharing the same letters are not significantly different from each other (*p* > 0.05) whatever the conditions.

Q-PCR quantifications of targeted bacterial groups showed that total bacteria concentrations were not affected by the 48 h feed deprivation stress nor the diet change (Figure 4A). The abundance of *Escherichia/Shigella* group increased significantly (*P* < 0.05) at day 9 and 15 in the bioreactor medium, when compared to day 7. In the mucin beads, the concentration of *Escherichia/Shigella* increased significantly (*P* < 0.05) at day 15 compared to day 9 and 11 for both (Figure 4B). PMA-qPCR confirmed that bacteria from *Escherichia/Shigella* genus were viable across time for all the fermentation runs in both the bioreactor and bead medium of the MPigut-IVM (data not shown).

**FIGURE 5:**
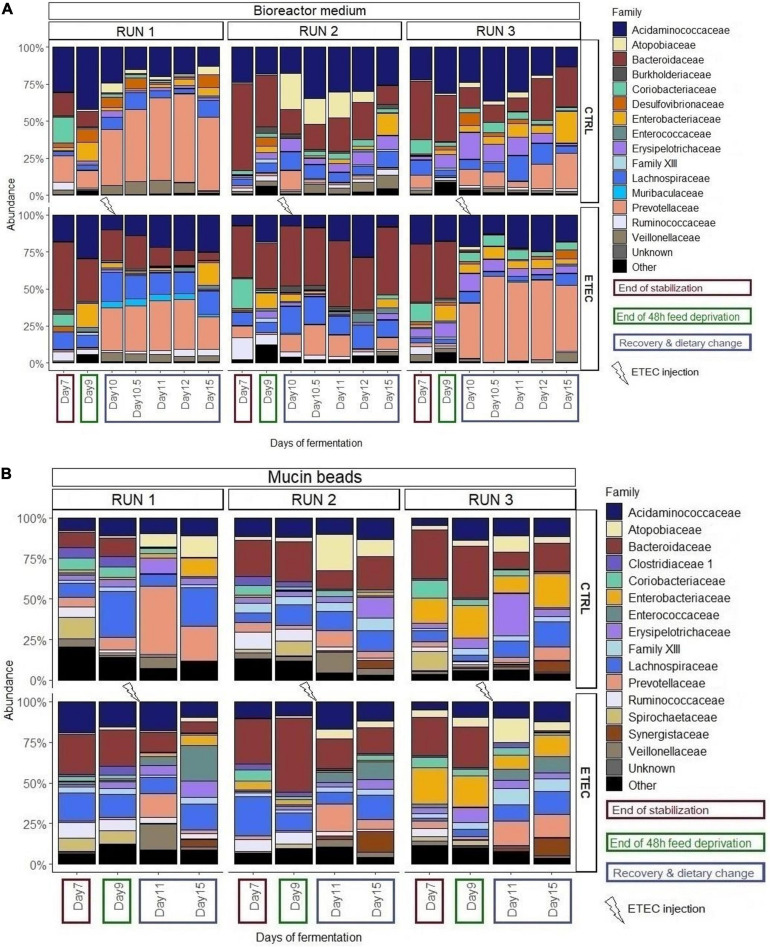
Relative abundances of the main bacterial families in the bioreactor medium **(A)** and the mucin beads **(B)** in MPigut-IVM during the runs #1, 2, and 3 which were subjected to a simulated weaning transition and challenged or not with the ETEC Ec105 strain.

**FIGURE 6:**
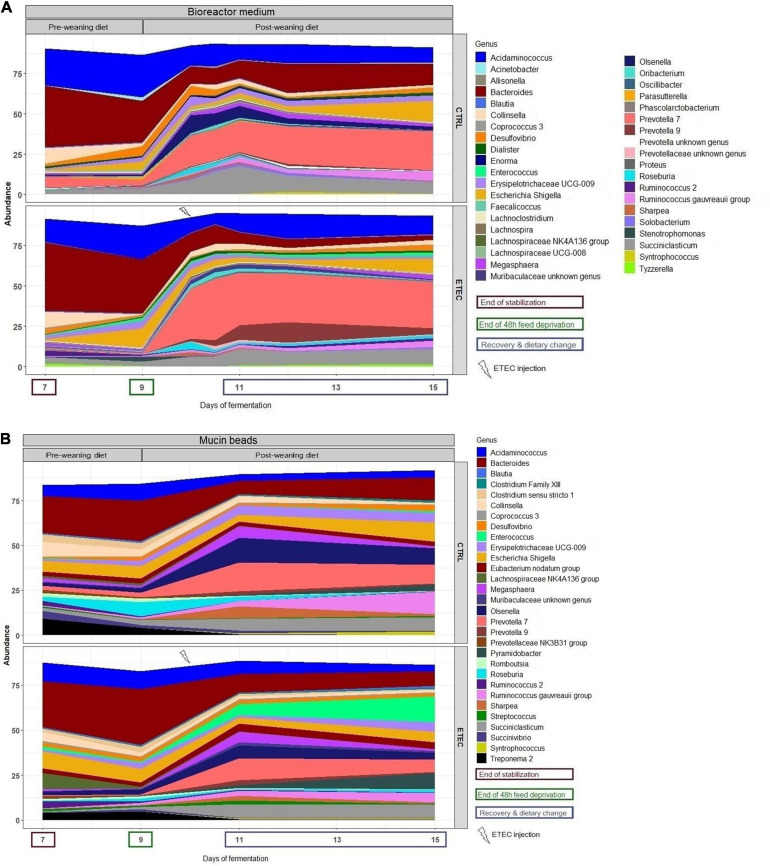
Mean relative abundance of the 30 more abundant bacterial genera in the bioreactor medium **(A)** and mucin beads **(B)** of MPigut-IVM in the CTRL and ETEC conditions (*n* = 3).

16S DNA sequencing analysis showed a certain variability of microbiota composition between runs #1, 2, and 3. However, several populations responded in a similar manner and were significantly impacted by the simulated weaning transition. In more details, on the mucin beads, the Spirochaetes phylum significantly decreased from a relative abundance of 6.8 ± 5.7% at day 7 to 0.3 ± 0.3% of mean relative abundance from day 10 to 15 (Supplementary Figure 2). In the bioreactor, the *Proteobacteria* significantly increased from a relative abundance of 4.0 ± 2.5% at day 7 to 17.1 ± 4.1% at day 9 and to 7.9 ± 5.6% from day 10 to 15 (Supplementary Figure 2). Looking at lower taxonomy level, some families were also significantly impacted by the simulation of weaning transition (Figure 5). In the bioreactor medium, *Coriobacteriaceae* significantly decreased from a mean relative abundance of 11.9 ± 6.6% at day 7 to a mean relative abundance of 2.2 ± 2% from day 9 to day 15. In contrast, the *Enterobacteriaceae* and *Erysipelotrichaceae* families significantly increased from a mean relative abundance of, respectively, 0.9 ± 0.7% and 1.5 ± 2% at day 7 to mean relative abundances of 6.2 ± 5.4% and 4.7 ± 4.1% from day 9 to day 15 (Figure 5A). Finally, the *Prevotellaceae* significantly decreased from 6.5 ± 6.3% at day 7 to 4 ± 2.7% at day 9 prior to significantly increase from day 10 to day 15 with a mean relative abundance of 27.1 ± 20%. On the mucin beads, the average relative abundances of *Bacteroidiaceae* and *Coriobacteriaceae* for the day 11–day 15 period (10.3 ± 8.8% and 1.3 ± 0.9%, respectively) significantly decreased as compared to those calculated for the day 7–day 9 period (24.9 ± 9% and 5 ± 2.7%, respectively) (Figure 5B). In contrast, the average relative abundances of *Prevotellaceae* and *Atopobiaceae* were significantly higher for the day 11–day 15 period (9.7 ± 7% and 6.8 ± 4%, respectively) than those calculated for the day 7–day 9 period (3.1 ± 2.4% and 2.4 ± 1.5%, respectively) (Figure 5B). The simulation of weaning transition also led to modifications in relative proportions of several genera, both in bioreactor medium and mucin beads (Figure 6). Statistical results of this section are detailed in Supplementary Table 6.

**FIGURE 7:**
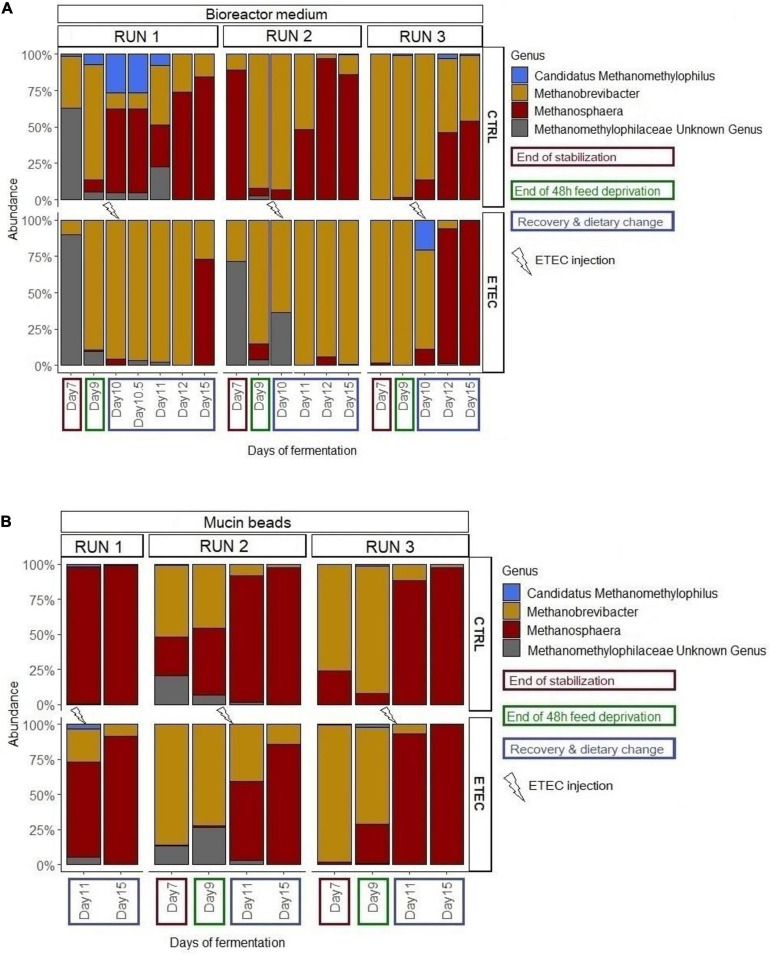
Relative abundance of the archaeal genera in the bioreactor medium **(A)** and mucin beads **(B)** of the MPigut-IVM for the runs #1, 2, and 3, in the CTRL and ETEC conditions. Day 7 and 9 of run #1 were removed due to their low number of sequences.

In the bioreactor medium and the mucin beads of control fermentations, the *Methanosphaera* genus was found in significantly higher mean relative abundance during the recovery period compared to the end of the stabilization period (Figure 7A), and feed deprivation stress also affected *Methanobrevibacter* relative abundance which was significantly lower after stress but only on mucin beads (Figure 7B). Regarding qPCR data, on the mucin beads, the abundance of archaea increased significantly (*P* < 0.05) at day 15 compared to day 9 and 11 for the CTRL conditions (Figure 4B).

### Survival of Ec105 in the MPigut-IVM

**FIGURE 8:**
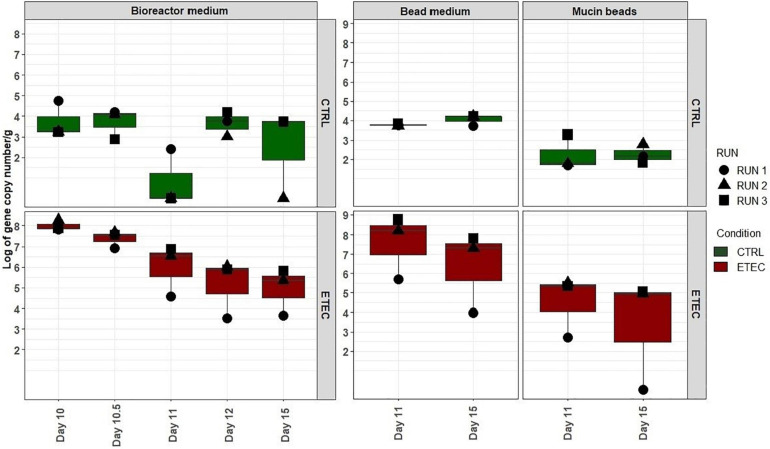
Quantification of the ETEC LT gene in the MPigut-IVM by qPCR for the runs #1, 2, and 3 in the CTRL and ETEC conditions (*n* = 3).

The presence of the ETEC strain was monitored in all the MPigut-IVM samples by quantifying the LT gene using qPCR. In the bioreactor medium of the ETEC condition, the LT gene was quantified at a mean of 8.0 ± 0.2 log_10_ of gene copy/g of sample at day 10 (ETEC inoculation) prior to slowly decrease over time to reach a mean of 4.9 ± 1.1 log_10_ of gene copy/g of sample at day 15 (end of recovery period). On the mucin beads of ETEC condition, the LT gene was quantified at mean values of 4.5 ± 1.6 and 3.3 ± 2.9 log_10_ of gene copy/g of sample, respectively, at day 11 and 15. However, the LT gene was quantified in higher concentration in the bead medium at mean values of 7.6 ± 1.6 and 6.4 ± 2.1 log_10_ of gene copy/g of sample, respectively, at day 11 and 15. It can be noticed that the LT gene was quantified in much lower quantity from day 11 in all samples from run #1 (Figure 8). Finally, a low copy number of LT gene could also be detected in the CTRL condition at mean values of 2.9 ± 1.6, 3.4 ± 1.6, and 3.9 ± 0.2 log_10_ of gene copy/g of sample, respectively, in the bioreactor medium, mucin beads, and bead medium, however, these values are close to the detection limit (Figure 8). The activity of the ETEC strain in the MPigut-IVM was evaluated by quantification of virulence gene expression using RT-qPCR. Even though the expression of virulence genes was detected in the bioreactor medium and bead medium in the ETEC condition on the day 10, 11, and 15, none of the targeted virulence genes (EAST1, LT, and K88 genes) was expressed in the CTRL condition (data not shown).

### Effects of the ETEC Ec105 Strain on MPigut-IVM Microbiota Metabolic Activity

Relative abundances of SCFAs were compared between the conditions ETEC and CTRL during the recovery period from day 10 to 15. The proportion of caproate and propionate tended (0.05 < *P* < 0.1) to be higher in the ETEC condition versus CTRL in the bead medium and bioreactor medium, respectively (Figure 2A).

The mucin bead medium metabolome shifted strongly between day 7 and day 11 in the presence of ETEC Ec105 (Figure 3). Indeed, the relative concentrations of isovalerate, valerate, 3-phenylpropionate and tyramine increased significantly, while the relative concentration of ethanol decreased. Multivariate PLS-DA analysis also suggested metabolome differences between CTRL and ETEC groups at day 11 (Figure 3). However, the relative concentrations of individual metabolites were not significantly different at this time point.

### ETEC Challenge Triggers Microbiota Composition Disruptions in the MPigut-IVM Subjected to a Simulated Weaning Transition

**FIGURE 9:**
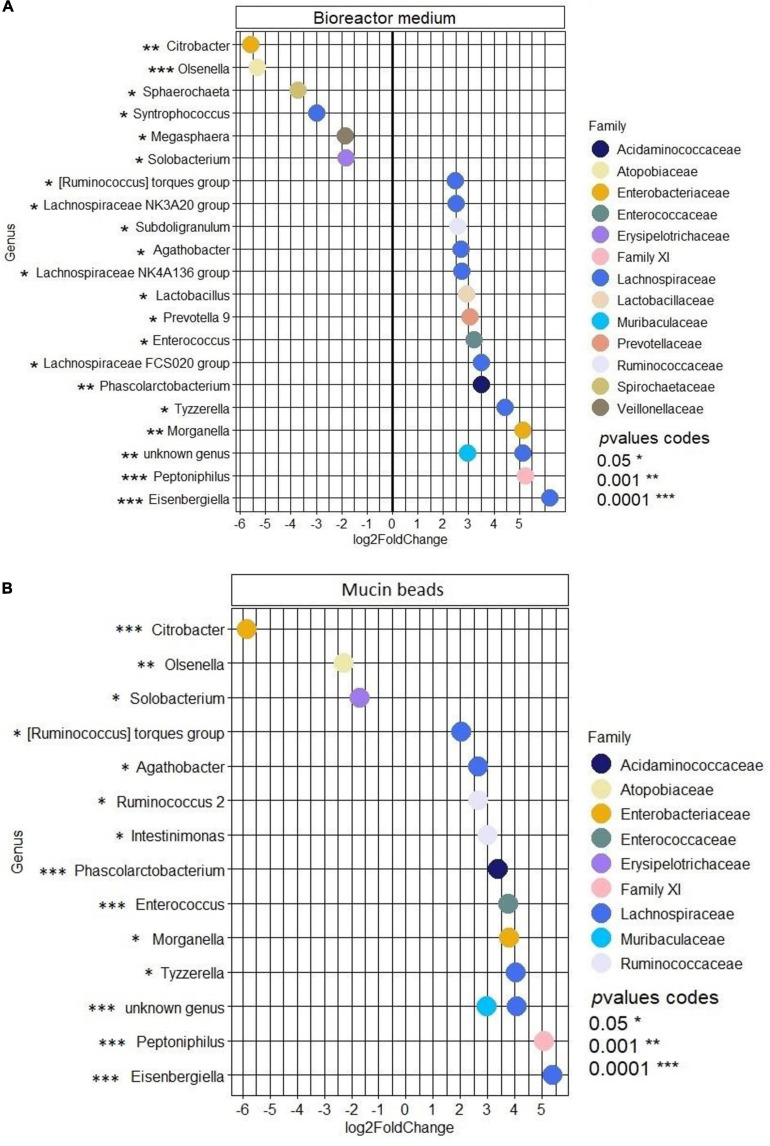
Differentially abundant genera between the samples of the recovery period containing the ETEC Ec105 strain or not in the bioreactor medium **(A)** and mucin beads **(B)** of the MPigut-IVM. Only significant Log2 fold changes are represented on the figure. Positive Log2 fold changes indicate genera that were significantly more abundant in the presence of the pathogen. *P* values were corrected for multiple testing.

No effect of the presence of the ETEC Ec105 strain on bacterial populations was detected by qPCR (Figure 4). However, a significant impact of the ETEC challenge on the microbiota was highlighted by the Illumina MiSeq data from day 10 to 15. Indeed, in the bioreactor medium, the presence of Ec105 led to significant higher average relative abundance of the Bacteroidetes phylum (35.4 ± 15.4% for the CTRL condition and 50.7 ± 10.5% for the ETEC condition for the day 10 to day 15 period), and lower mean relative abundance of Actinobacteria from 9.2 ± 7.4% in the CTRL condition to 2.7 ± 2.7% in the ETEC condition during the same period (Supplementary Figure 2A). On the mucin beads, the Firmicutes were significantly increased from 48.9 ± 7.7% in the CTRL condition to 58.1 ± 6.6% in the ETEC condition from day 11 to 15 (Supplementary Figure 2B). At the family level in the bioreactor medium (Figure 5A), the *Prevotellaceae* average relative abundance was significantly increased (23.2 ± 23% in the CTRL condition, 30.9 ± 18.8% in the ETEC condition for the day 10 – day 15 period) (Figure 5A). In contrast, *Acidaminococcaceae*, *Atopobiaceae*, *Veillonellaceae*, and *Erysipelotrichaceae* were significantly reduced with average relative abundances of, respectively, 24.2 ± 8.4%, 6.3 ± 7%, 4.1 ± 2.8%, and 6 ± 4.8% in the CTRL condition to 18.2 ± 7.4%, 0.3 ± 0.4%, 2.2 ± 2.1%, and 2.7 ± 2.8% in the ETEC condition during the same recovery period (Figure 5A). On the mucin beads, the *Atopobiaceae* family was significantly decreased from mean relative abundance of 11.5 ± 3.2% in the CTRL condition to 5.8 ± 2.1% in the ETEC condition while the *Enterococcaceae* was significantly increased from 0.5 ± 0.6% in the CTRL condition to 10.3 ± 5.7% in the ETEC condition in the day 11 – day 15 period (Figure 5B). At the genus level, the introduction of the ETEC strain significantly affected the abundance of several genera. For example, the genera *Eisenbergiella, Peptoniphilus, Morganella, Tyzzerella*, and *Enterococcus* were particularly enhanced in the ETEC-challenged bioreactors and mucin beads (Figures 6, 9). The MPigut-IVM archaeal microbiota was not significantly impacted by ETEC inoculation (Figure 7).

The MPigut-IVM microbiota alpha diversity indices were not significantly modified by any of the treatment (Supplementary Figure 4). Yet, beta diversity analysis using principal component analysis on Bray-Curtis distance showed that samples of MPigut-IVM clustered by diet (Supplementary Figure 4). No effect of ETEC was detected (data not shown).

### Gene Expression in IPI-2I Cells Is Modulated When Exposed to the Bead Medium of MPigut-IVM Challenged by ETEC

**FIGURE 10:**
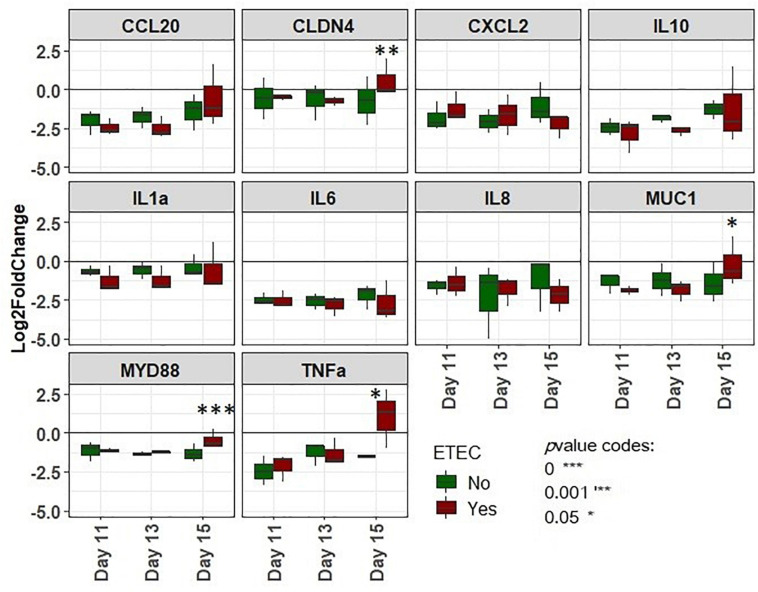
Log2 fold changes of gene expression of IPI-2I cells incubated with bead medium supernatants of the MPigut-IVM collected at days 11, 13, and 15 and challenged or not with the ETEC Ec105 strain. Values were normalized with basal gene expression profiles for the targeted genes from IPI-2I cells incubated with their usual glutamine and FCS complemented DMEM medium (*n* = 3).

The expression of selected genes involved in innate inflammatory immune response targeting inflammatory cytokines and chemokines, tight junctions or mucus secretion (Supplementary Table 5) of IPI-2I porcine cells incubated with filtrated effluents of the bead medium of MPigut-IVM were quantified using RT-qPCR (Figure 10). The effluents from the ETEC-inoculated bioreactors collected at day 15 led to a significant increase in the expression of TNFα, MYD88, MUC1, and CLDN4 genes compared to effluents from control bioreactors collected at same day. Differences in IPI-2I gene expression caused by effluents from the other days of fermentation (day 11 and 13) remained non-significant.

## Discussion

The MPigut-IVM has been developed to simulate the gut microbiome and mucobiome of weaning piglet (Gresse et al., 2021). In this previous paper, we demonstrated that, due to unique features such as the presence of mucin beads and a self-maintained anaerobiosis, the MPigut-IVM harbored an *in vitro* microbiota very close to that of the proximal colon of piglets originating from the same farm, after a stabilization period of 7 days (Gresse et al., 2021). To further understand the impact of weaning stressors on the colonic microbiota of piglets and their roles in the etiology of post-weaning diarrhea, a diet change focused on the introduction of more diversified plant protein sources and of higher amounts of these nutrients was introduced into the MPigut-IVM right after the 48 h- feed deprivation period to evaluate the impact of stressful events close to weaning as encountered in commercial pig farms. First, in the present work, the simulated weaning transition induced modifications in the archaeal microbiota of the MPigut-IVM, shifting from a *Methanobrevibacter* to a *Methanosphaera* dominant microbiota. Modifications of the archaeal microbiota associated with weaning transitions are not yet well documented in piglets. This shift could, however, impact the metabolites present in the piglet gut, considering the high abundance of archaea populations in piglet lower gut (Gresse et al., 2019). Indeed, *Methanobrevibacter* and *Methanosphaera* are mainly known as hydrogenotrophic and methylotrophic archaea genera, respectively, although recent genomic studies suggest that they may have other metabolic differences (Poehlein et al., 2018). Second, regarding bacterial microbiota, the simulated weaning transition induced an increase in the relative abundance of several bacterial communities in the MPigut-IVM, including *Prevotellaceae* and *Enterobacteriaceae* family members while the *Bacteroidiaceae* was decreased. Our findings are in agreement with *in vivo* studies reporting a shift from a high relative abundance of *Bacteroides* in pre-weaning piglet feces toward a high relative abundance of *Prevotella* coupled with a reduced *Bacteroides* proportion in post-weaning piglet fecal samples (Alain B. Pajarillo et al., 2014; Frese et al., 2015; Guevarra et al., 2018; Trckova et al., 2018; Yang et al., 2019) and Adhikari et al. (2019) reported similar results in the piglet colon. *Prevotella* is a common commensal genus which plays important roles in the digestion of nutrients, particularly in the degradation of starch, proteins and other plant polysaccharides (Ivarsson et al., 2014; Sandberg et al., 2019). In the study from Yang et al. (2019), *Prevotella* was the most abundant genus in both healthy and diarrheic weaning piglets. However, piglets that developed diarrhea after weaning harbored a higher relative abundance of *Prevotella* and less *Escherichia coli* in their pre-weaning period compared to piglets that remained healthy after weaning (Yang et al., 2019). A reduced number of *Bacteroides* in weaned piglet feces was also associated with diarrhea (Yang et al., 2019). Therefore, disturbed ratios between *Prevotella*, *Bacteroides*, and *Escherichia* populations in early life could be associated with the onset of post-weaning diarrhea. Also, the simulated weaning transition in the MPigut-IVM led to a decrease of the *Collinsella* genus from the *Coriobacteriaceae* family. A decreased proportion of *Collinsella* in human gut was previously linked to the development of a dysbiotic microbiota in inflammatory bowel disease (Kassinen et al., 2007) while an increase of *Collinsella* was positively correlated with protection against rotavirus diarrhea in gnotobiotic pigs (Twitchell et al., 2016) suggesting of potential role of *Collinsella* in pigs’ health. In our study, microbial activity was also significantly modified by the simulated weaning transition probably associated to microbiota composition changes. Redox potential was considerably impacted by the feed deprivation period which is in agreement with our previous findings (Gresse et al., 2021). In the bioreactor and on the mucin beads, butyrate, acetate and isovalerate relative abundances significantly decreased after the simulated weaning transition. In contrast, propionate, valerate and caproate increased both in the bioreactor and on the mucin beads of the MPigut-IVM. The total SCFA concentration increased during the feed deprivation period. This particular point could be due to the accumulation of these metabolites inside the bioreactors due the absence of feeding and consequently medium flushing. Besides, the bacterial metabolite 3-phenylpropionate increased after weaning simulation in the mucin bead medium, maybe due to an increased availability of its polyphenol precursors present in the post-weaning diet. Butyrate proved to be very beneficial to piglet health by improving the performance of piglets around weaning due to the stimulation of intestinal epithelium (Lu et al., 2008), improved immune response (Melo et al., 2016), and modulation of intestinal microbiota (Castillo et al., 2006; Xu et al., 2016; López-Colom et al., 2019). Also, supplementation of early weaned piglets with sodium butyrate was shown to attenuate diarrhea symptoms and decrease intestinal permeability (Feng et al., 2018). Thus, fluctuations of both bacterial composition and activity were observed in the MPigut-IVM following the simulated weaning transition which could favor the emergence of opportunistic pathogens such as ETEC.

To further simulate the conditions leading to post-weaning diarrhea at weaning (Gresse et al., 2017), a porcine ETEC strain was inoculated to the MPigut-IVM after the simulated weaning transition. Interestingly, 2 days after the inoculation of ETEC Ec105, the Labile Toxin (LT) gene was detected in high concentration in the bead medium of the MPigut-IVM, showing a preferential localization of ETEC Ec105 close to an area rich in mucins which are indeed known to be a privileged site of adhesion *in vivo* (González-Ortiz et al., 2014). Only one replicate displayed a strong decrease in the pathogenic strain concentration after few days in the MPigut-IVM. This difference could be explained by some variability in the microbial composition of the fecal inocula, probably reflecting the *in vivo* situation. A more robust microbial community could lead to less susceptibility to the introduction of a pathogen. The differences of microbiota composition observed between the three replicates could also induces different responses toward the ETEC challenge and the simulated weaning transition such as observed *in vivo*. The ETEC challenge did not influence the relative abundance of *Escherichia/Shigella* genus, probably because the concentration of the ETEC strain after inoculation was in the range of that of commensal *Escherichia/Shigella*. In addition, López-Colom et al. (2019) reported previously that an ETEC challenge was not associated with the increase of commensal *E. coli* in the ileum and ileal mucosa of weaned piglets. In this study, piglets which received butyrate and heptanoate preventive treatments to ETEC F4 displayed higher levels of commensal Enterobacteria in the ileum and ileal mucosa few days after the infection (López-Colom et al., 2019). One hypothesis, supported by Leatham et al. (2009), was the phenomenon of colonization resistance described as an increase in coliforms in the gut to fight against colonization of pathogenic strains. In the present work, several families and genera were, however, significantly impacted by the introduction of ETEC after the simulated weaning transition in the MPigut-IVM. The most spectacular change was the increase in Enterococcus in the ETEC conditions, especially on the mucin beads. Some *Enterococcus* members are known to bind to mucus *in vivo* (Hendrickx et al., 2015; Tytgat et al., 2016). Consistently with our findings, several *in vivo* studies reported a higher relative abundance or quantity of *Enterococcus* and *E. coli* or ETEC strains in the digestive content or mucosa of neonatal diarrheic piglets (Cheon and Chae, 1996; Jonach et al., 2014; Larsson et al., 2014; Hermann-Bank et al., 2015). A co-occurrence of *Enterococcus* and pathogenic *E. coli* could thus be involved in the pathogenesis of diarrhea episodes in piglets. Considering the several reports of this observation, it has been hypothesized that *Enterococcus* and *E. coli*/ETEC members could be able to naturally cooperate possibly by mechanisms of cross feeding or mutualism (Germerodt et al., 2016). Finally, the *Morganella* genus, which was found to be four times more abundant in the ETEC condition both in the mucin beads and the bioreactor by the differential analysis, has been previously associated with diarrhea in humans (Müller, 1986; Jertborn and Svennerholm, 1991; Ikeobi et al., 1996) and was even positively correlated with diarrhea indices during an ETEC F4 challenge in mice (Xu et al., 2020). The MPigut-IVM is thus able to reproduce several characteristics of ETEC pathogenesis consistent with *in vivo* data. In consequence, the other populations which correlated with the ETEC conditions but are not currently described in the literature such as *Peptoniphilus* or *Eisenbergiella* should be considered as populations of interest for future investigations.

To increase our understanding of the etiology of post-weaning diarrhea and how microbiota disruptions and pathogen presence affect host cells, we incubated diluted filtrated effluents collected from bead medium from MPigut-IVM with the IPI-2I pig intestinal cell line. *In vitro* models of the human colon were previously successfully coupled with eukaryotic cell cultures (Marzorati et al., 2014; Defois et al., 2018) but, to our knowledge, this is the first time that this was performed with an *in vitro* model of the piglet colon. In this study, supernatants from the bead medium of the ETEC condition induced significant increases in expression of genes coding for the myeloid differentiation primary response 88 (MyD88), tumor necrosis factor α (TNFα), claudin 4 (CLDN4), and mucin 1 (MUC1) in IPI-2I cells. The relation between mucins, commensals and pathogens have been widely studied (Etienne-Mesmin et al., 2019). MUC1 is a high molecular mass glycoprotein expressed at the apical surface of mucosal epithelial cells. MUC1 secretion can be stimulated by lipopolysaccharides produced by Gram negative bacteria and mucin is thought to play an important role against infections of epithelial cells (Kato et al., 2017). Expression of the MUC1 gene notably limited the access of Helicobacter pylori in infected mice (McGuckin et al., 2007). In our study, claudin 4 gene was over-expressed in cells exposed to supernatants collected at day 15 of ETEC conditions. This gene belongs to claudins’ family which are, with occludin proteins, tight junction proteins located at the apical side of piglet enterocytes (Pasternak et al., 2015) and thus play a role in intestinal permeability and infection. Consistently with our findings, claudin protein higher gene expression was previously associated with ETEC K88 infection in the porcine cell line IPEC-J2 in several studies (Wu et al., 2016; Luo et al., 2019). We could hypothesize that the higher expression of claudin genes from enterocytes could be induced in order to repair disrupted tight junctions due to the presence of toxins or other virulence factors in the supernatants which, as reported by RT-qPCR, were indeed expressed in the bead medium of the MPigut-IVM. The MyD88-mediated innate immune response has been already proven to be primarily important for protection against microbial pathogen infection via the induction of inflammatory cytokine production (Scanga et al., 2002; Campos et al., 2004; von Bernuth et al., 2012; Issac et al., 2013, 2018). Indeed, MyD88 deficient mice showed to be profoundly susceptible to infection (Issac et al., 2013). In weaning piglets, an increased level of MyD88 was already reported in enterocytes both *in vivo* and *in vitro* by several studies after challenge with an ETEC K88 strain (Chytilová et al., 2014; Finamore et al., 2014; Xu et al., 2014). TNFα is one of the most widely studied proinflammatory cytokine involved in numerous bacterial, parasitic and viral infections and was suggested as a biomarker of digestive pathologies in weaned piglets due to its correlation with villi/crypt ratio damages (Van Reeth et al., 2002; Gustavo Hermes et al., 2013; Barba-Vidal et al., 2017; López-Colom et al., 2019). Indeed, increased concentration of TNFα or increased expression of the associated gene were previously reported in the intestine of piglets orally challenged with ETEC K88 (Pu et al., 2018; López-Colom et al., 2019) as well as in IPEC-J2 cells co-incubated with ETEC K88 (Xia et al., 2017a, b). Considering the matches between our results and literature, the incubation of MPigut-IVM bead medium supernatants with IPI-2I cells seemed to reproduce, at least in part, the effects of ETEC infection on piglet enterocytes. However, it is important to notice that significant differences were detected only with effluent collected at day 15. These findings could highlight that the introduction of a porcine ETEC strain in the MPigut-IVM led to modifications of microbial metabolites which could be secreted in sufficient quantity to induce differential inflammatory responses in porcine epithelial cells at day 15 only. The high concentration of tyramine and valerate or the low concentration of ethanol in the mucin bead medium after weaning simulation in the ETEC group could contribute to the transcriptomic regulation observed in epithelial cells *in vitro*.

To conclude, our studies reported that the reproduction of a weaning transition in the MPigut-IVM impacted the microbiota composition and functionality in a consistent manner compared to previous *in vivo* findings. The introduction of a porcine ETEC strain after the simulated weaning transition was positively correlated with an increase in several genera which should be considered in future investigations. If further research needs to be undertaken to fully understand the interactions, our results confirmed that members of *Prevotella*, *Escherichia*, and *Enterococcus* genera could play a role in the onset of post-weaning diarrhea in piglets. The incubation of the *in vitro* effluents with pig intestinal cell lines, which was performed for the first time, indicated that MPigut-IVM was able to report some effect of microbiota metabolites change on host cells. The MPigut-IVM seems to reproduce the effects of weaning transition and ETEC colonization on the microbiota and will thus be used to evaluate preventive strategies against post-weaning intestinal dysbiosis.

## Data Availability Statement

The datasets presented in this study can be found in online repositories. The names of the repository/repositories and accession number(s) can be found below: https://www.ncbi.nlm.nih.gov/, BioProject ID PRJNA703771.

## Ethics Statement

Ethical review and approval was not required for the animal study because we collected only fecal material.

## Author Contributions

RG, FC-D, EF, and SB-D: conceptualization. RG, SD, FC-D, EF, TV, AJ-M, JJG, and SB-D: methodology. RG, AJ-M, and MB: formal analysis. RG: writing – original draft preparation. RG, FC-D, SD, EF, TV, MB, and SB-D: writing – review and editing. RG and MB: visualization. FC-D, EF, JJG, and SB-D: supervision. All authors read and approved the final manuscript.

## Conflict of Interest

FC-D and RG are employees of Lallemand SAS. The authors declare that this study received funding from Lallemand SAS. The funder had the following involvement in the study: study design, data analysis, interpretation of the data and writing of the article. The remaining authors declare that the research was conducted in the absence of any commercial or financial relationships that could be construed as a potential conflict of interest.

## Footnotes

1 http://sigenae-workbench.toulouse.inra.fr

2 www.R-project.org

3 http://genex.gene-quantifcation.info/

## Supplementary Material

Supplementary Figure 1 Analyses of the pooled fecal inocula used for runs #1, 2 and 3: relative abundance of SCFAs measured by gas chromatography **(A)**, relative abundances of the principal phyla **(B)**, and families **(C)** measured by 16S Illumina sequencing.

Supplementary Figure 2 Relative abundances of the main bacterial phyla in the bioreactor medium **(A)** and the mucin beads **(B)** in MPigut-IVM during the runs 1, 2, and 3.

Supplementary Figure 3 Shannon **(A)** and observed **(B)** alpha diversity indexes MPigut-IVM samples collected from runs #1, 2 and 3 (*n* = 3).

Supplementary Figure 4 Principal Component analysis (PCoA) plot with Bray-Curtis dissimilarity on the bacterial communities separated by the type of diet in the bioreactor medium, bead medium, and mucin beads of the MPigut-IVM.
