# Supplementary figures and images for: Pathogen Challenge and Dietary Shift Alter Microbiota Composition and Activity in a Mucin-Associated in vitro Model of the Piglet Colon (MPigut-IVM) Simulating Weaning Transition
Source: Front Microbiol. 2021 Jul 19;12:703421. doi: 10.3389/fmicb.2021.703421 (PMC8328230; doi:10.3389/fmicb.2021.703421)

**A**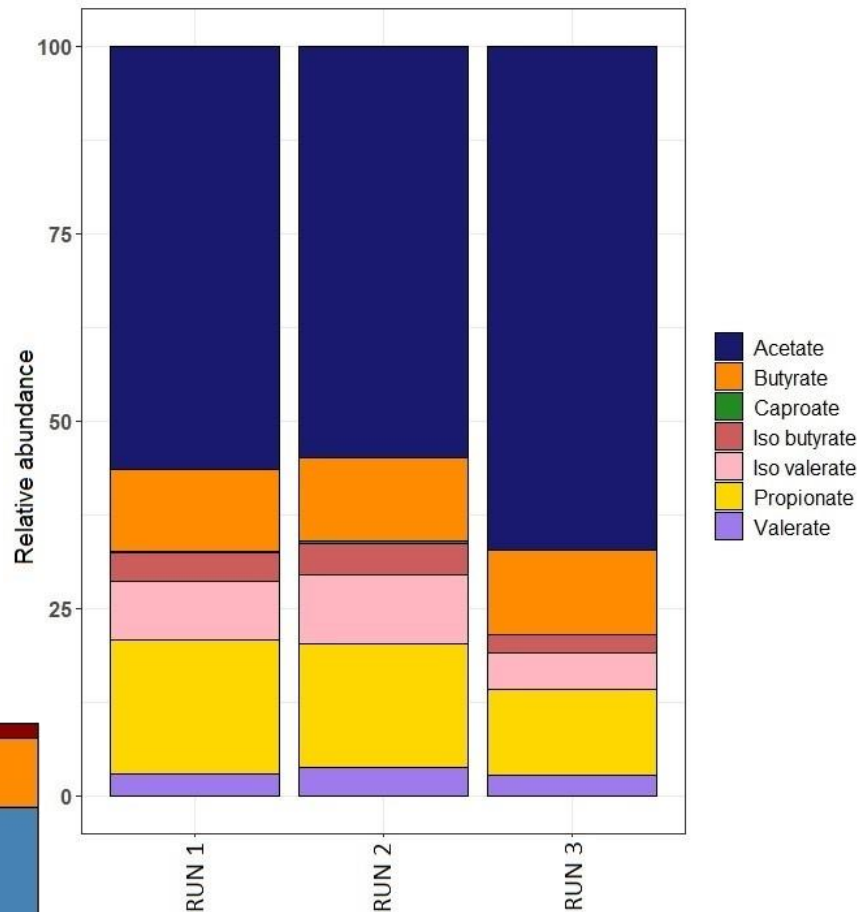**B**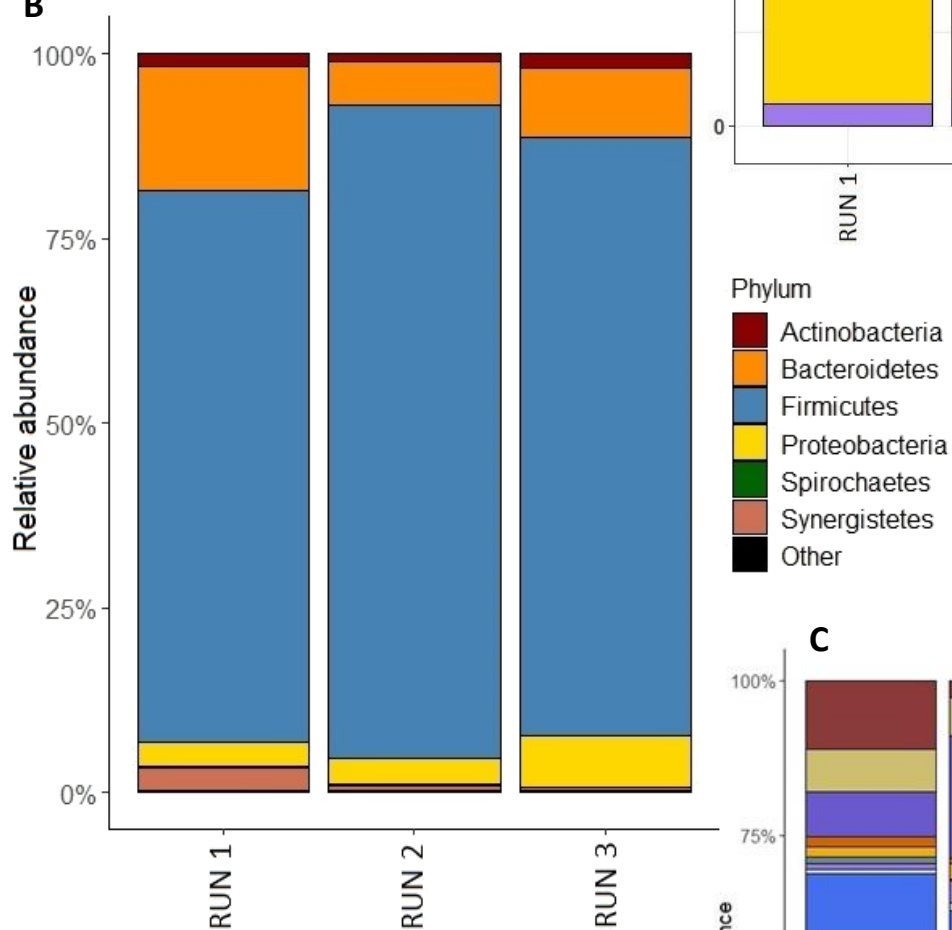**C**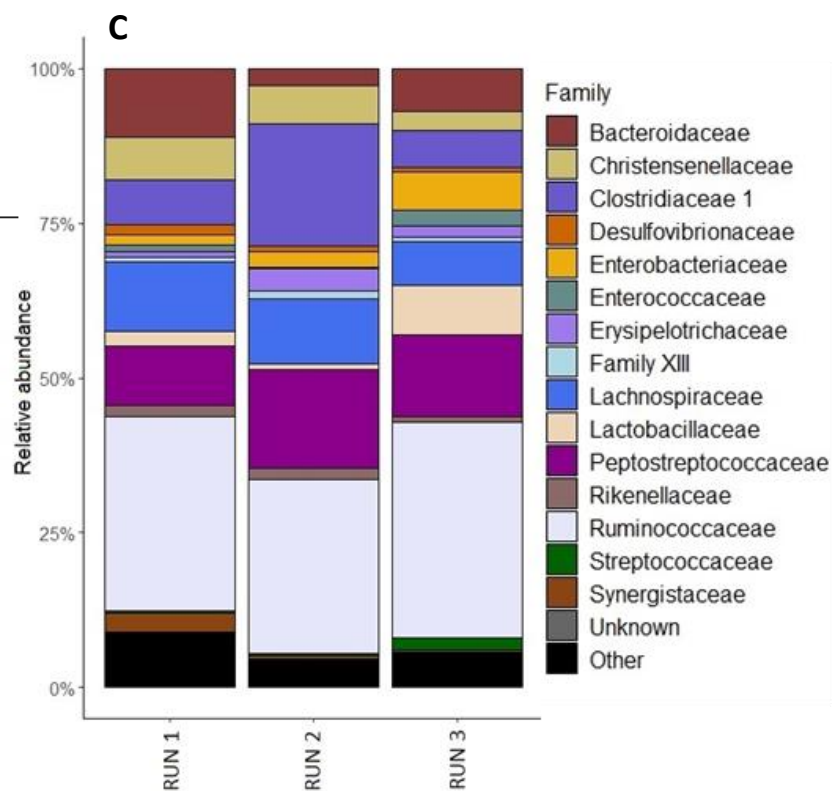

Supplement: Supplementary Figure 1 — Analyses of the pooled fecal inocula used for runs #1, 2 and 3: relative abundance of SCFAs measured by gas chromatography (A), relative abundances of the principal phyla (B), and families (C) measured by 16S Illumina sequencing. [file Image_1.pdf]

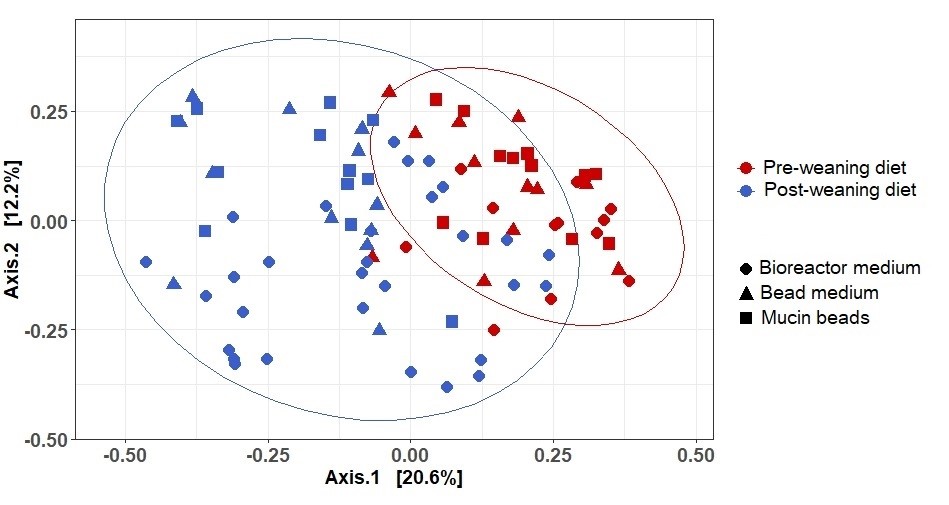

Supplement: Supplementary Figure 4 — Principal Component analysis (PCoA) plot with Bray-Curtis dissimilarity on the bacterial communities separated by the type of diet in the bioreactor medium, bead medium, and mucin beads of the MPigut-IVM. [file Image_4.jpg]
