# Supplementary material for: Pathogen Challenge and Dietary Shift Alter Microbiota Composition and Activity in a Mucin-Associated in vitro Model of the Piglet Colon (MPigut-IVM) Simulating Weaning Transition
Source: Front Microbiol. 2021 Jul 19;12:703421. doi: 10.3389/fmicb.2021.703421 (PMC8328230; doi:10.3389/fmicb.2021.703421)
Supplement: Supplementary Figure 2 — Relative abundances of the main bacterial phyla in the bioreactor medium (A) and the mucin beads (B) in MPigut-IVM during the runs 1, 2, and 3. [file Image_2.pdf]

A

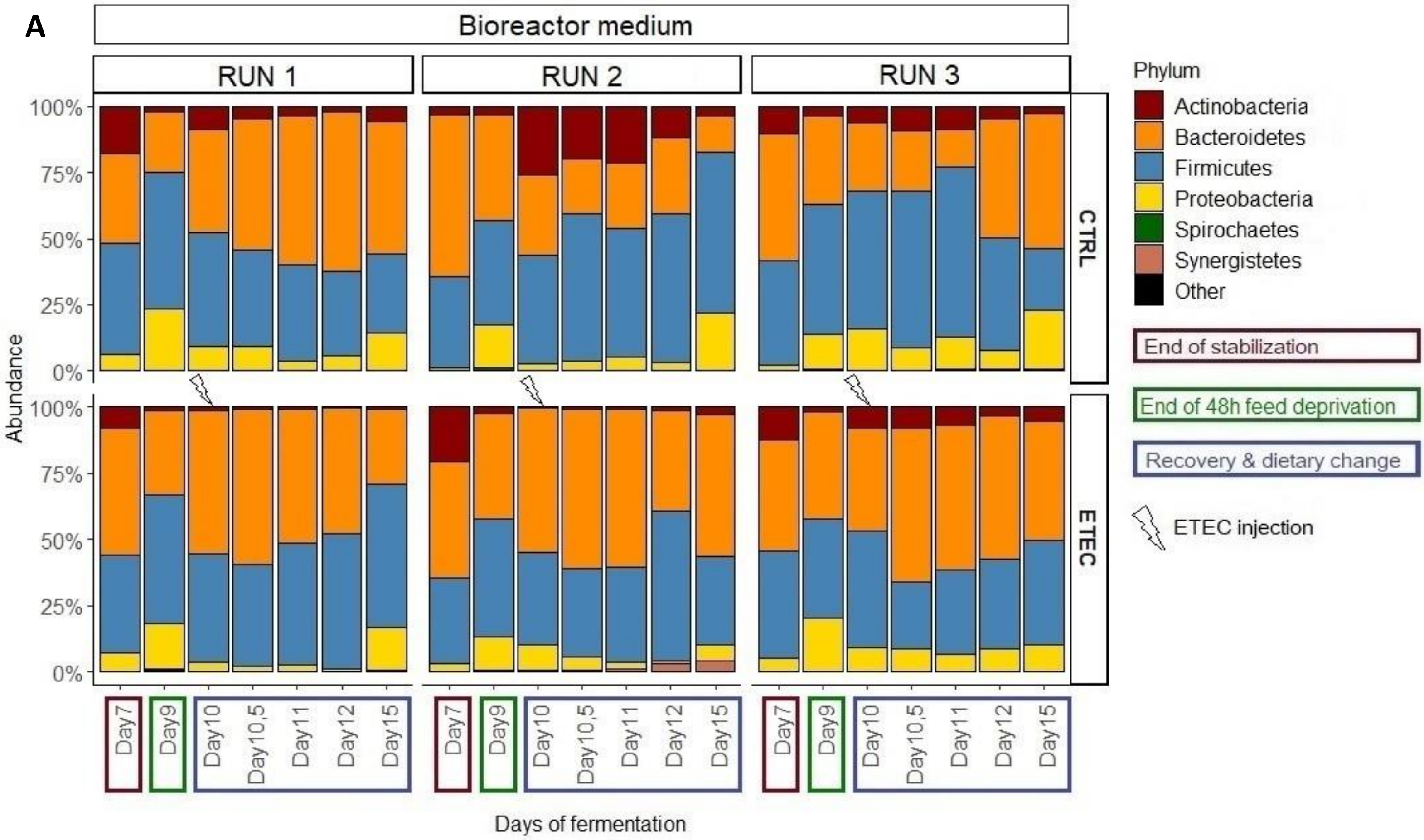

Bioreactor medium
RUN 1
RUN 2
RUN 3
CTRL
ETEC
Abundance
100%
75%
50%
25%
0%
Day7
Day9
Day10
Day10,5
Day11
Day12
Day15
Days of fermentation
Phylum
Actinobacteria
Bacteroidetes
Firmicutes
Proteobacteria
Spirochaetes
Synergistetes
Other
End of stabilization
End of 48h feed deprivation
Recovery & dietary change
ETEC injection

B

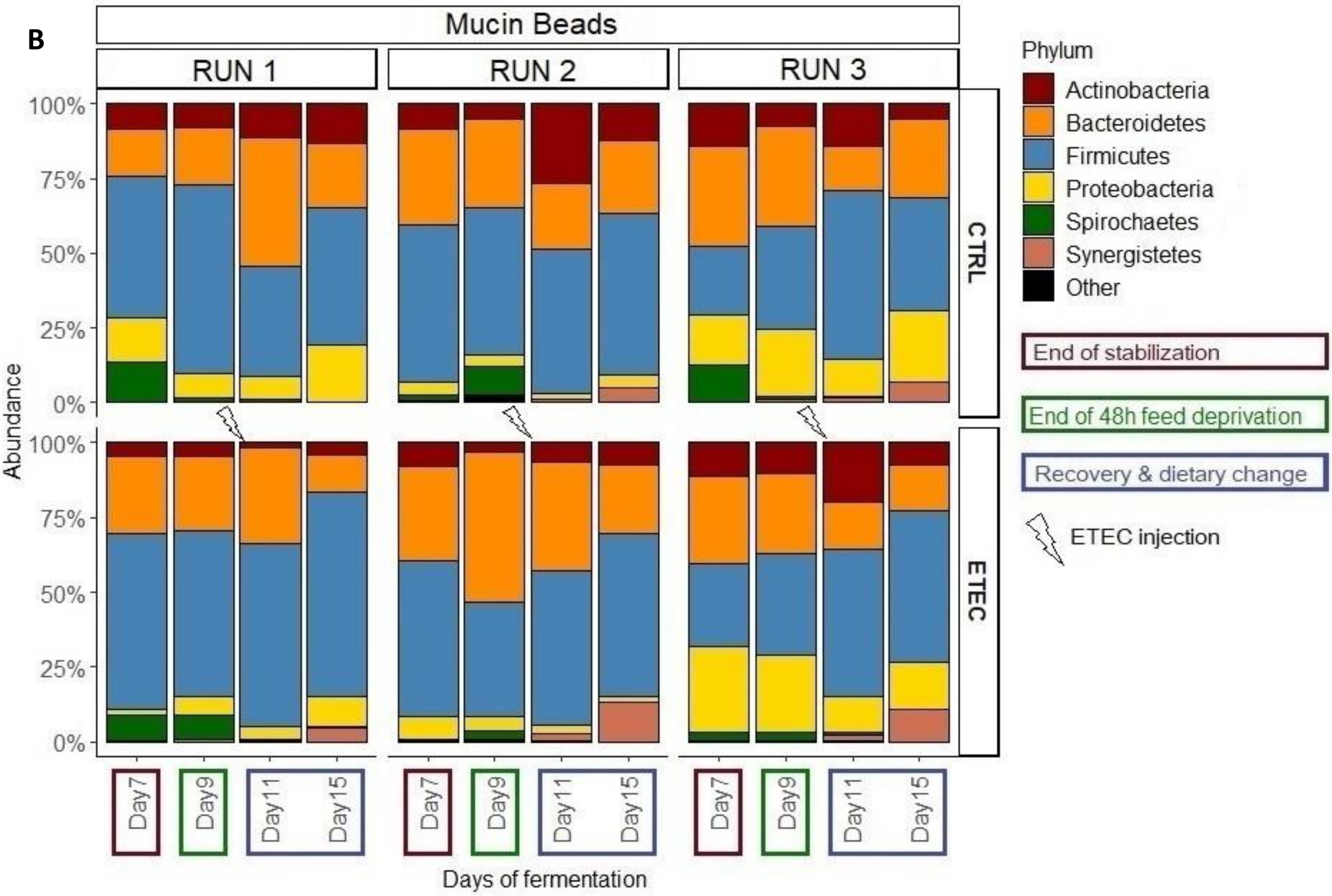

Mucin Beads
RUN 1
RUN 2
RUN 3
CTRL
ETEC
Abundance
100%
75%
50%
25%
0%
Day7
Day9
Day11
Day15
Days of fermentation
Phylum
Actinobacteria
Bacteroidetes
Firmicutes
Proteobacteria
Spirochaetes
Synergistetes
Other
End of stabilization
End of 48h feed deprivation
Recovery & dietary change
ETEC injection
