# Supplementary material for: Pathogen Challenge and Dietary Shift Alter Microbiota Composition and Activity in a Mucin-Associated in vitro Model of the Piglet Colon (MPigut-IVM) Simulating Weaning Transition
Source: Front Microbiol. 2021 Jul 19;12:703421. doi: 10.3389/fmicb.2021.703421 (PMC8328230; doi:10.3389/fmicb.2021.703421)
Supplement: Supplementary Figure 3 — Shannon (A) and observed (B) alpha diversity indexes MPigut-IVM samples collected from runs #1, 2 and 3 (n = 3). [file Image_3.pdf]

A

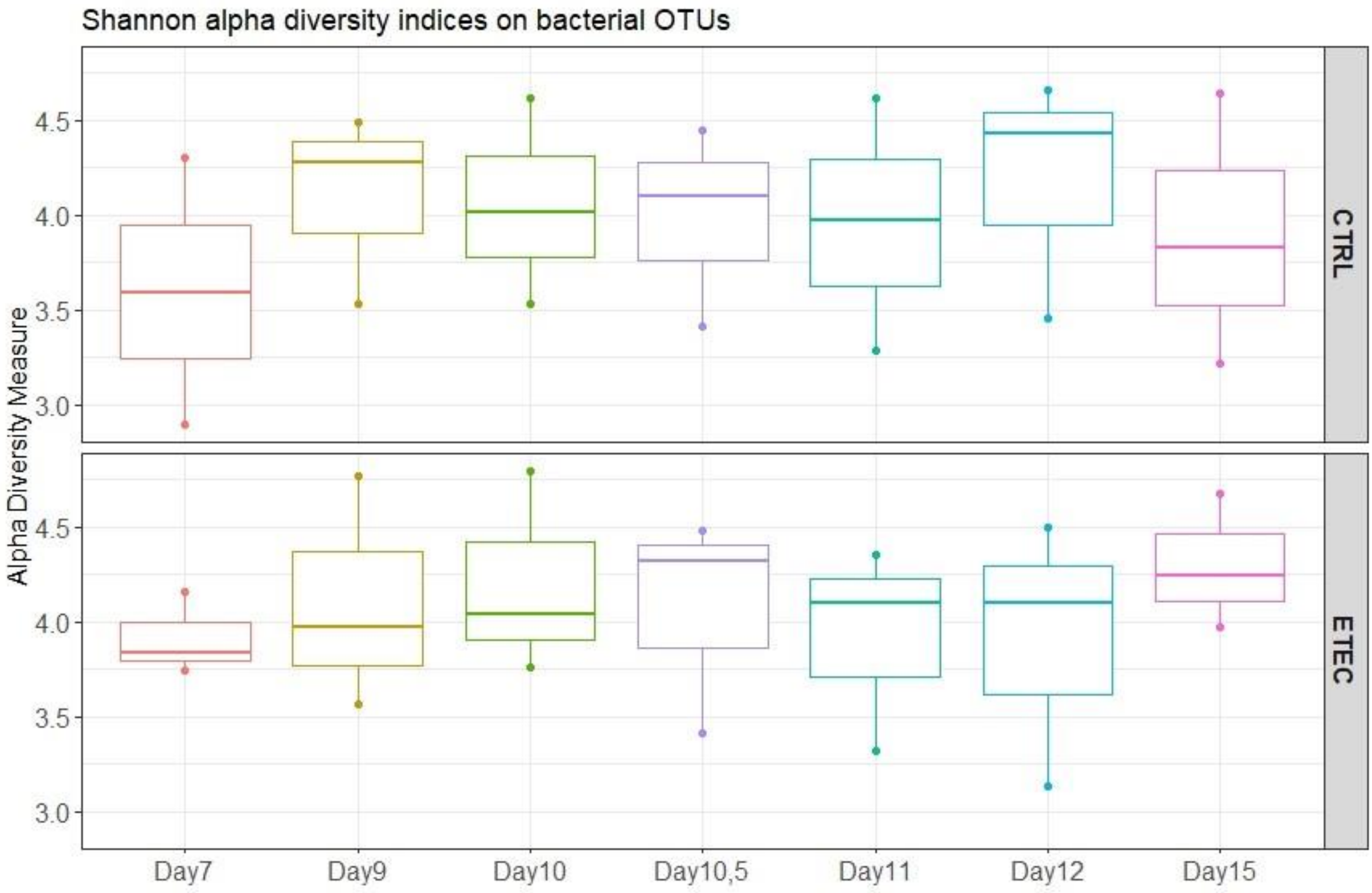

Shannon alpha diversity indices on bacterial OTUs
Alpha Diversity Measure
CTRL
ETEC
4.5
4.0
3.5
3.0
Day7
Day9
Day10
Day10,5
Day11
Day12
Day15

B

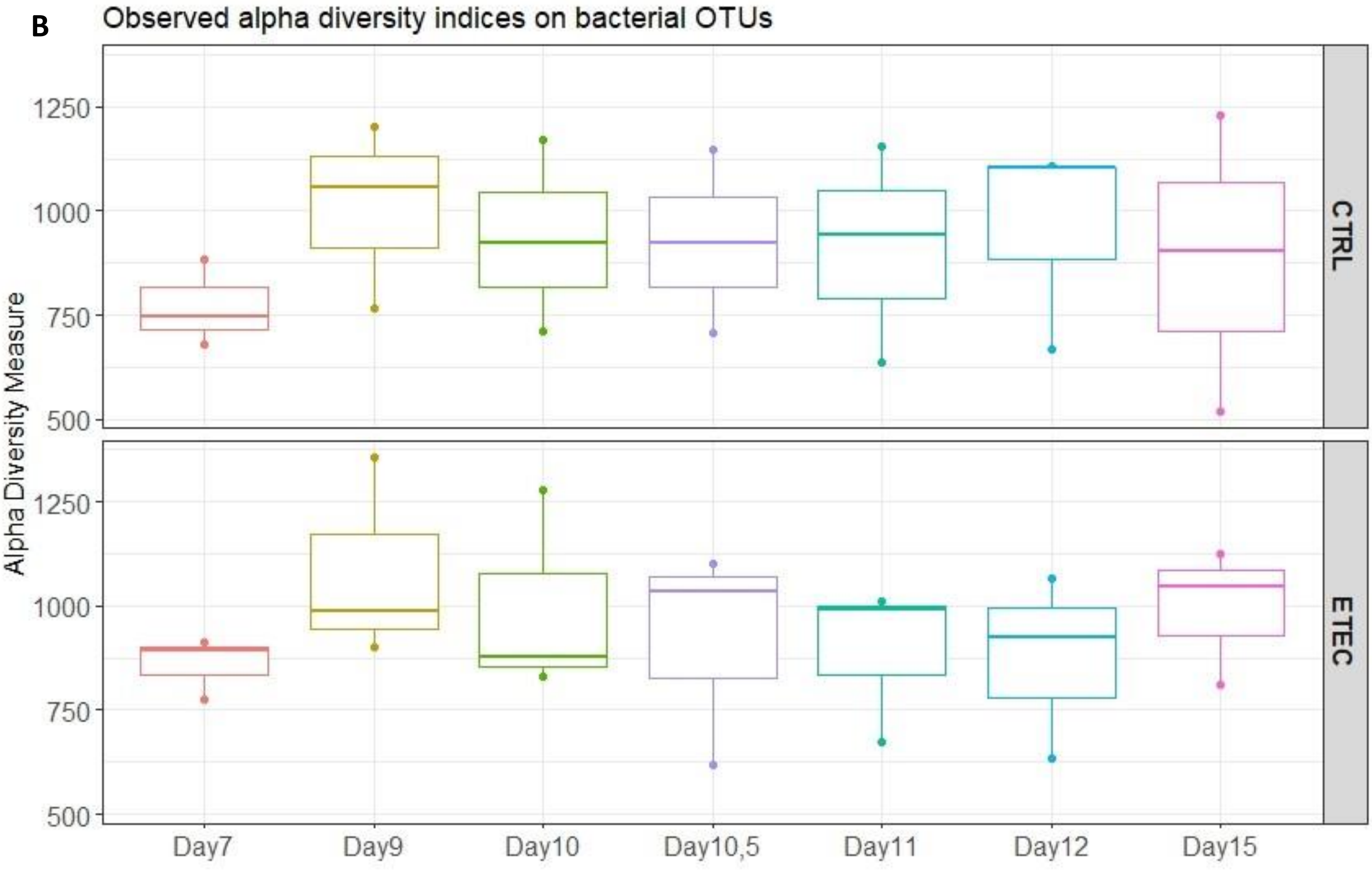

Observed alpha diversity indices on bacterial OTUs
Alpha Diversity Measure
CTRL
ETEC
1250
1000
750
500
Day7
Day9
Day10
Day10,5
Day11
Day12
Day15
