# Supplementary material for: Pathogen Challenge and Dietary Shift Alter Microbiota Composition and Activity in a Mucin-Associated in vitro Model of the Piglet Colon (MPigut-IVM) Simulating Weaning Transition
Source: Front Microbiol. 2021 Jul 19;12:703421. doi: 10.3389/fmicb.2021.703421 (PMC8328230; doi:10.3389/fmicb.2021.703421)
Supplement: Supplementary file 5 [file Table_1.DOCX]

**Supplementary table 1.** Composition of the media fed to the bioreactor and mimicking ileal chyme of 28 days old piglets corresponding to the pre and post-weaning diet.

|  | Medium mimicking  Pre-weaning diet | Medium mimicking  Post-weaning diet |
| --- | --- | --- |
| Concentration | g/L | g/L |
| Wheat starch | 5 | 5 |
| Corn starch | 0 | 6 |
| Pectin | 2 | 2 |
| Arabinogalactan | 2 | 2 |
| Cellulose | 2 | 3 |
| Soy protein isolate | 10 | 12 |
| Peptone from potatoes | 0 | 5 |
| Tryptone from casein | 4.5 | 1.5 |
| Mucin from porcine stomach type II | 2 | 2 |
| Whey powder | 5 | 2 |
| FeSO_4_, 7H_2_O | 0.005 | 0.005 |
| L-cysteine HCl monohydrate | 0.80 | 0.80 |
| Porcine bile extract | 0.40 | 0.40 |
| KH_2_PO_4_ | 0.5 | 0.5 |
| NaHCO_3_ | 1.5 | 1.5 |
| Hemin from porcine | 0.005 | 0.005 |
| NaCl | 4.5 | 4.5 |
| KCl | 4.5 | 4.5 |
| MgSO_4_ anhy. (120.37 g mol^-1^) | 0.64 | 0.64 |
| CaCl_2_ 2H_2_O (147.02 g mol^-1^) | 0.15 | 0.15 |
| MnCl_2_ 4H_2_O (197.91 g mol^-1^) | 0.20 | 0.20 |

**Supplementary table 2.** Primers and probes used for quantification of various bacteria and methanogenic archaea.

| Target | Type | Sequences | References | Concentration | Annealing temperature |
| --- | --- | --- | --- | --- | --- |
| *Escherichia coli*/ *Shigella* group | Taqman | 5’-CAT GCC GCG TGT ATG AAG AA-3’  5’-CGG GTA ACG TCA ATG AGC AAA-3’  (6-FAM)-5’-TAT TAA CTT TAC TCC CTT CCT CCC CGC TGA A-3’(TAMRA) | Huijsdens et al. | 300 nM  300 nM  100 nM | 61°C |
| Total Bacteria | SYBRGREEN | 5’-ACT CCT ACG GGA GGC AG-3’  5’-GTA TTA CCG CGG CTG CTG-3’ | Yu et al. | 500 nM  500 nM | 61°C |
| Methanogenic archaea | SYBRGREEN | 5’-GAG GAA GGA GTG GAC GAC GGTA-3’  5’-ACG GGC GGT GTG TGC AAG-3’ | Ohene-adjei et al. | 500 nM  500 nM | 61°C |
| Labile enterotoxin (LT) | SYBRGREEN | 5’- GGC GTT ACT ATC CTC TCT AT  3’—TGG TCT CGG TCA GAT ATG T | Madoroba et al. | 500 nM  500 nM | 55°C |

**Supplementary table 3. Primers used for quantification of selected ETEC virulence genes expression.**

| **Target** | **Gene** | **Sequence 5’ 3’** | **Concentration nM** | **Reference** |
| --- | --- | --- | --- | --- |
| ST toxin B | *Stb* | F- TGCCTATGCATCTACACAAT | 150 | Madoroba et al. |
|  |  | R- CTCCAGCAGTACCATCTCTA | 150 |  |
| Labile enterotoxin | *LT* | F- GGCGTTACTATCCTCTCTAT | 500 | Madoroba et al |
|  |  | R- TGGTCTCGGTCAGATATGT | 500 |  |
| Fimbriae 4 | *K88* | F- GAATCTGTCCGAGAATATCA | 200 | Madoroba et al |
|  |  | R- GTTGGTACAGGTCTTAATGG | 200 |  |
| Enteroaggregative heat stable enterotoxin 1 | *EAST1* | F- TCGGATGCCATCAACACAGT | 500 | Ngeleka et al. |
|  |  | R- GTCGCGAGTGACGGCTTTGTAG | 500 |  |
| Sigma 38 protein | *rpos* | F- GCGCGGTAGAGAAGTTTGAC | 500 | Rahman et al. |
|  |  | R- GGCTTATCCAGTTGCTCTGC | 500 |  |
| Aerobic control respiration | *arcA* | F- GTTCTTACCCGGCAGATTGA | 500 | Roussel et al. |
|  |  | R- CAGACCCCGCACATTCTTAT | 500 |  |
| GAPDH *E. coli* | *gapA* | F- CGTTGAAGTGAAAGACGGTCATC | 200 | Nicklasson et al. |
|  |  | R- CAACACCAACTTCGTCCCATTT | 200 |  |

**Supplementary table 4:** Metabolites identified by NMR metabolomics in bead medium. *: indicates the peak used for quantification based on the corresponding bucket intensity (not overlapping with peaks from other metabolites). Multiplicity of signals is indicated within brackets: s, singlet; d, doublet; t, triplet; m, multiplet.

|  | Metabolite | δ^1^H (ppm) |
| --- | --- | --- |
| 1 | 2-methylbutyrate | 0.86 (t), 1.39* (m) |
| 2 | Valerate | 0.89 (t), 1.31* (m), 1.53 (m), 2.19 (t) |
| 3 | Butyrate | 0.90* (t), 1.56 (m), 2.16 (t) |
| 4 | Isovalerate | 0.91 (d), 1.96 (m), 2.06* (d) |
| 5 | Isoleucine | 0.94* (t), 1.01 (d) |
| 6 | Leucine | 0.97* (t) |
| 7 | Valine | 1.00* (d), 1.05 (d), 3.62 (d) |
| 8 | Propionate | 1.06* (t), 2.19 (m) |
| 9 | Isobutyrate | 1.07 (d), 2.40* (m) |
| 10 | Ethanol | 1.19* (t), 3.66 (m) |
| 11 | 5-aminovalerate | 1.65 (m), 2.24* (t), 2.33 (t), 3.02 (t) |
| 12 | Cadaverine | 1.73* (m), 3.02 (t) |
| 13 | Putrescine | 1.78* (m), 3.05 (t) |
| 14 | Acetate | 1.92* (s) |
| 15 | p-cresol | 2.26 (s), 6.84* (d), 7.15 (d) |
| 16 | 3-(4-hydroxyphenyl)propionate | 2.45* (t), 2.82 (t), 6.86 (d), 7.18 (d) |
| 17 | 3-phenylpropionate | 2.50* (t), 2.89 (t), 7.27 (t), 7.32 (d), 7.37 (t) |
| 18 | Methylamine | 2.60* (s) |
| 19 | Trimethylamine | 2.88* (s) |
| 20 | Tyramine | 2.94 (t), 3.24 (t), 6.92* (d), 7.23 (d) |
| 21 | Methanol | 3.36* (s) |
| 22 | Phenylacetate | 3.54* (s), 7.31 (t), 7.39 (t) |
| 23 | Formate | 8.46* (s) |

**Supplementary table 5. Primer used for quantification of gene expression in IPI-2I cells exposed to MPigut-IVM supernatants.**

| **Target** | **Gene** | **Sequence 5’ 3’** | **Annealing temperature** | **Reference** |
| --- | --- | --- | --- | --- |
| Cyclophilin A | *Cyclophilin-A* | CCT GAA CAT ACG GGT CCT G | 57 | Dr. J.J. Garrido |
|  |  | AAC TGG GAA CCG TTT GTG TTG |  |  |
| Beta actin | *β-actin* | CAGGTCATCACCATCGGCAACG | 57 | Dr. J.J. Garrido |
|  |  | GACAGCACCGTGTTGGCGTAGAGGT |  |  |
| Chemokine (C-C motif) ligand 20 | *CCL20* | ACTT TGA CTG CTG CCT CCG ATA | 54 | Dr. J.J. Garrido |
|  |  | TGC ATT GAT GTC ACA AGC TTC A |  |  |
| Claudin 4 | *CLDN4* | TAT CAT CCT GGC CGT GCT A | 57 | 42 |
|  |  | CAT CAT CCA CGC AGT TGG T |  |  |
| Chemokine (C-X-C motif) ligand 2 | *CXCL2* | GGA TAG CAC GCT GTA CCA TC | 57 | Dr. J.J. Garrido |
|  |  | ACT GTC TCA ATA AAT AAC AAC CGA C |  |  |
| Interleukin 10 | *IL10* | CAG ATG GGC GAC TTG TTG | 57 | Dr. J.J. Garrido |
|  |  | ACA GGG CAG AAA TTG ATG AC |  |  |
| Interleukin 1a | *IL1a* | AAC GAA GAC GAA CCC GTG TTG CT | 57 | Dr. J.J. Garrido |
|  |  | GGT CTC ATC TTT GAT GGT TTT GG |  |  |
| Interleukin 6 | *IL6* | TGG CTA CTG CCT TCC CTA CC | 57 | Dr. J.J. Garrido |
|  |  | CAG AGA TTT TGC CGA GGA TG |  |  |
| Interleukin 8 | *IL8* | TTC GAT GCC AGT GCA TAA ATA | 57 | Dr. J.J. Garrido |
|  |  | CTG TAC AAC CTT CTG CAC CCA |  |  |
| Mucin 1 | *MUC1* | CCC TGG CCA TCA TCT ATG TC | 56 | 42 |
|  |  | TGC CCA CAG TTC TTT CGT C |  |  |
| Myeloid differentiation primary response 88 | *MYD88* | TGG TGG TGG TTG TCT CTG ATG A | 57 | Dr. J.J. Garrido |
|  |  | TGG AGA GAG GCT GAG TGC AA |  |  |
| Tumor Necrosis Factor alpha | *TNF-α* | CGC CCA CGT TGT AGC CAA TGT | 57 | Dr. J.J. Garrido |
|  |  | CAG ATA GTC GGG CAG GTT GAT CTC |  |  |

**Supplementary table 6:** Statistical analysis of the principal bacterial or archaeal phyla families or genera detected by Ilumina sequencing in the bioreactor (A) and on the mucin beads (B). Statistical analyses were performed using linear mixed-models with time point (days of fermentation) as a fixed effect and fermentation experiment as a random effect. Means associated with a different letter are significantly different. P adj : adjusted p-values (FDR method).

**A**

|  | pval | padj | 7 | 9 | 10 | 10.5 | 11 | 12 | 15 |
| --- | --- | --- | --- | --- | --- | --- | --- | --- | --- |
| Methanobrevibacter | 0.158 | 0.21 | a | a | a | a | a | a | a |
| Methanosphaera | 0.013 | 0.025 | ab | ab | ab | ab | b | ab | a |
| Archaea unknown genus | 0.001 | 0.003 | a | a | a | a | a | b | a |
| Candidatus Methanomethylophilus | 0.259 | 0.259 | a | a | a | a | a | a | a |
| Bacteroidaceae | 0.001 | 0.003 | a | a | a | a | a | b | ab |
| Acidaminococcaceae | 0.366 | 0.499 | a | a | a | a | a | a | a |
| Prevotellaceae | 0.005 | 0.011 | a | a | a | a | a | a | a |
| Lachnospiraceae | 0.098 | 0.163 | a | a | a | a | a | a | a |
| Enterobacteriaceae | 0 | 0 | ab | ab | ab | ab | c | a | bc |
| Erysipelotrichaceae | 0.018 | 0.036 | b | ab | ab | ab | ab | a | ab |
| Coriobacteriaceae | 0 | 0 | a | a | a | a | a | b | a |
| Veillonellaceae | 0.546 | 0.546 | a | a | a | a | a | a | a |
| Atopobiaceae | 0.399 | 0.499 | a | a | a | a | a | a | a |
| Desulfovibrionaceae | 0.459 | 0.51 | a | a | a | a | a | a | a |
| Bacteroides | 0.001 | 0.003 | a | a | a | a | a | b | ab |
| Prevotella.7 | 0.028 | 0.046 | a | a | a | a | a | a | a |
| Acidaminococcus | 0.002 | 0.005 | a | a | a | ab | a | ab | b |
| Succiniclasticum | 0.003 | 0.006 | ab | ab | b | ab | ab | a | a |
| Escherichia Shigella | 0 | 0 | ab | ab | ab | a | c | a | bc |
| Collinsella | 0 | 0 | a | a | a | a | a | b | a |
| Desulfovibrio | 0.455 | 0.592 | a | a | a | a | a | a | a |
| Ruminococcus gauvreauii group | 0.006 | 0.011 | ab | ab | ab | ab | b | a | a |
| Megasphera | 0.859 | 0.859 | a | a | a | a | a | a | a |
| Roseburia | 0 | 0 | b | ab | a | a | a | a | a |
| Dialister | 0.58 | 0.686 | a | a | a | a | a | a | a |
| Prevotella.9 | 0.451 | 0.592 | a | a | a | a | a | a | a |
| Lachnoclostridium | 0.745 | 0.807 | a | a | a | a | a | a | a |
| Firmicutes | 0.839 | 0.839 | a | a | a | a | a | a | a |
| Bacteroidetes | 0.808 | 0.839 | a | a | a | a | a | a | a |
| Proteobacteria | 0 | 0 | a | a | a | a | b | a | b |
| Actinobacteria | 0.069 | 0.171 | a | a | a | a | a | a | a |
| Synergistetes | 0.309 | 0.514 | a | a | a | a | a | a | a |

**B**

|  | pval | padj | 7 | 9 | 11 | 15 |
| --- | --- | --- | --- | --- | --- | --- |
| Methanobrevibacter | 0 | 0 | a | a | b | b |
| Methanosphaera | 0 | 0 | b | b | a | a |
| Archeae unknown genus | 0.077 | 0.102 | a | a | a | a |
| Candidatus Methanomethylophilus | 0.249 | 0.249 | a | a | a | a |
| Bacteroidaceae | 0 | 0 | a | a | b | b |
| Lachnospiraceae | 0.187 | 0.229 | a | a | a | a |
| Acidaminococcaceae | 0.533 | 0.555 | a | a | a | a |
| Prevotellaceae | 0.003 | 0.006 | b | ab | a | a |
| Enterobacteriaceae | 0.076 | 0.119 | a | a | a | a |
| Erysipelotrichaceae | 0.173 | 0.229 | a | a | a | a |
| Atopobiaceae | 0.003 | 0.006 | b | ab | a | a |
| Clostridium Family XIII | 0.555 | 0.555 | a | a | a | a |
| Coriobacteriaceae | 0 | 0 | a | a | b | a |
| Ruminococcaceae | 0 | 0 | ab | a | c | b |
| Enterococcaceae | 0.028 | 0.052 | ab | b | ab | a |
| Escherichia Shigella | 0.129 | 0.161 | a | a | a | a |
| Prevotella.7 | 0.013 | 0.02 | b | ab | a | a |
| Ruminococcus gauvreauii.group | 0 | 0.001 | a | b | a | a |
| Megasphaera | 0.001 | 0.003 | b | a | a | a |
| Desulfovibrio | 0.526 | 0.552 | a | a | a | a |
| Eubacterium nodatum group | 0.552 | 0.552 | a | a | a | a |
| Bacteroides | 0 | 0 | a | a | b | b |
| Ruminococcus.2 | 0 | 0 | a | a | b | a |
| Prevotella.9 | 0.013 | 0.02 | b | ab | ab | a |
| Acidaminococcus | 0.013 | 0.02 | ab | a | ab | b |
| Lachnospiraceae.NK4A136.group | 0.269 | 0.311 | a | a | a | a |
| Romboutsia | 0 | 0 | a | a | b | b |
| Erysipelotrichaceae.UCG.009 | 0.089 | 0.121 | a | a | a | a |
| Collinsella | 0 | 0 | a | a | b | a |
| Olsenella | 0.003 | 0.006 | b | ab | a | a |
| Firmicutes | 0.179 | 0.224 | a | a | a | a |
| Bacteroidetes | 0.232 | 0.232 | a | a | a | a |
| Proteobacteria | 0.026 | 0.066 | a | ab | b | ab |
| Actinobacteria | 0.115 | 0.191 | a | a | a | a |
| Spirochaetes | 0 | 0.002 | a | a | b | ab |
